# Precision Dosing in Presence of Multiobjective Therapies by Integrating Reinforcement Learning and PK‐PD Models: Application to Givinostat Treatment of Polycythemia Vera

**DOI:** 10.1002/psp4.70012

**Published:** 2025-05-05

**Authors:** Alessandro De Carlo, Elena Maria Tosca, Paolo Magni

**Affiliations:** ^1^ Electrical, Computer and Biomedical Engineering University of Pavia Pavia Italy

**Keywords:** adaptive dosing protocol, AI/ML, Givinostat, model‐informed precision dosing, pharmacometrics, PK‐PD modeling, precision medicine, reinforcement learning

## Abstract

Precision dosing aims to optimize and customize pharmacological treatment at the individual level. The integration of pharmacometric models with Reinforcement Learning (RL) algorithms is currently under investigation to support the personalization of adaptive dosing therapies. In this study, this hybrid technique is applied to the real multiobjective precision dosing problem of givinostat treatment in polycythemia vera (PV) patients. PV is a chronic myeloproliferative disease with an overproduction of platelets (PLT), white blood cells (WBC), and hematocrit (HCT). The therapeutic goal is to simultaneously normalize the levels of these efficacy/safety biomarkers, thus inducing a complete hematological response (CHR). An RL algorithm, Q‐Learning (QL), was integrated with a PK‐PD model describing the givinostat effect on PLT, WBC, and HCT to derive both an adaptive dosing protocol (QL_pop_‐agent) for the whole population and personalized dosing strategies by coupling a specific QL‐agent to each patient (QL_ind_‐agents). QL_pop_‐agent learned a general adaptive dosing protocol that achieved a similar CHR rate (77% vs. 83%) when compared to the actual givinostat clinical protocol on 10 simulated populations. Treatment efficacy and safety increased with a deeper dosing personalization by QL_ind_‐agents. These QL‐based patient‐specific adaptive dosing rules outperformed both the clinical protocol and QL_pop_‐agent by reaching the CHR in 93% of the test patients and completely avoided severe toxicities during the whole treatment period. These results confirm that RL and PK‐PD models can be valid tools for supporting adaptive dosing strategies as interesting performances were achieved in both learning a general set of rules and in customizing treatment for each patient.

**Summary**

- What is the current knowledge on the topic?
  ○ The integration of Pharmacometric models with Reinforcement Learning (RL) algorithms to deliver precision dosing in realistic case studies with multiple biomarkers to optimize is still limited.
- What question did this study address?
  ○ This simulation study addresses the potentialities of RL and PK‐PD modeling in supporting adaptive dosing strategies and aims to answer these questions. What are the advantages of using an RL‐based personalized protocol in a complex clinical setting to guide the pharmacological treatment? How much do RL individualized adaptive dosing strategy performances differ from those of a clinical protocol? Can we use RL to learn a general adaptive dosing strategy? To this end, givinostat treatment in polycythemia vera patients was considered as a demonstrative case study.
- What does this study add to our knowledge?
  ○ RL was used to learn both a general adaptive dosing protocol in a population and personalized dosing rules tailored to each patient. RL learned from scratch a general protocol with similar performances to that of clinical experts. RL‐based personalized dose strategies outperformed the two general protocols in maximizing all clinical outcomes in the presence of large IIV. Although further extensions and evaluations are still necessary to refine the RL treatment personalization, this work provides a useful starting point to set up the methodology.
- How might this change drug discovery, development, and/or therapeutics?
  ○ Pharmacometric models can support precision dosing. From their integration with RL, it is possible to derive personalized dose strategies that can increase the efficiency of pharmacological treatments.

## 1 Introduction

Precision dosing refers to tailoring the dose of a drug or a drug combination in order to maximize the benefit/risk balance for each patient [1, 2, 3]. It has been recommended for several classes of compounds with a small therapeutic index and/or high interindividual variability (IIV), for example, antimicrobial, anticancer, biologics, antiretrovirals, or psychotropic agents [1, 2, 4]. Precision dosing is mainly applied during clinical practice [3, 5, 6], but it can also be exploited during drug development to manage the presence of high IIV, thereby reducing attrition rates [7]. Adaptive dosing strategies allow for delivering precision dosing in a time‐varying therapy setting (e.g., chronic diseases or progressive disease conditions), which requires periodical patient evaluations and, accordingly, dose adjustments [8]. In this context, drug plasma concentration (i.e., therapeutic drug monitoring, TDM [9]) and/or biomarkers of both drug efficacy and toxicity are monitored and used to adjust the dose level. Therefore, adaptive dosing rules are formalized as if‐then‐else statements depending on the monitored quantities.

Pharmacokinetic and Pharmacodynamic (PK‐PD) modeling and simulation play a central role in enabling precision dosing [5, 10]. The integration of these in silico methods within individual‐oriented pharmacology is called Model‐Informed Precision Dosing (MIPD) [3, 11, 12]. It combines population PK‐PD models with Bayesian estimation techniques to obtain the individual model parameters for each patient starting from collected data (i.e., drug concentrations or biomarker measurements). Then, the individual response to different dosing scenarios is simulated, enabling the selection of the most appropriate dosing strategy [11, 12].

The recent emergence of Artificial Intelligence (AI) and Machine Learning (ML) methods in Pharmacometrics [13, 14, 15] represents an opportunity to further improve the MIPD framework. In particular, Reinforcement Learning (RL) gained momentum for solving precision dosing problems [7, 16, 17]. RL is a branch of ML in which a computer (i.e., agent) is trained to learn an optimal policy that associates the most proper action to achieve a targeted condition with each system state [18]. The key to learning the optimal policy relies on an iterative agent–system interplay [18]. At each interaction step, the agent tries different actions and evaluates their consequences, observing the system evolution (i.e., next state). The system provides feedback, quantifying the suitability of the action performed in a certain condition through a numeric score (i.e., reward). The reward signal is central because it formalizes the agent's goal on the system and drives the learning of the optimal policy, which allows for maximizing the collected scores at each interaction.

The RL framework well fits the decision‐making process behind a precision dosing problem based on an adaptive dosing strategy as the agent adapts its choices by using the current evidence (i.e., system state monitoring) [16, 17, 19]. Consequently, the RL‐agent can be mapped with the clinician and the system with the patient. In the context of precision dosing, the experience needed to train an RL‐agent can be found in retrospective clinical datasets. However, it is difficult to find a great variety of dosing scenarios in real clinical repositories [20, 21]. To overcome this issue, different works have highlighted the importance of integrating population PK‐PD models within the RL‐framework [16, 19, 22, 23]. Indeed, model simulations compensate for the lack of real‐world experience, thus allowing a better training of RL‐agents based on several simulated dosing scenarios, including those harmful. This hybrid approach combining RL and PK‐PD models has already been explored to optimize different treatments [24, 25, 26, 27] especially in the oncology field [16, 22]. For example, frameworks based on a single RL‐agent were applied to derive a general adaptive dosing protocol for chemotherapy, able to optimize the dynamics of tumor size [28, 29, 30] or neutrophil count [31] in a population of patients. More recently, this paradigm was extended to individually personalize adaptive anticancer treatments on single patients, considering one efficacy/toxicity endpoint [31, 32]. Each patient was associated with a personal RL‐agent that was trained on a patient‐specific PK‐PD model. However, oncological treatments are characterized by a high degree of complexity as there is the need to simultaneously optimize multiple efficacy and/or toxicity endpoints.

In this work, a hybrid approach integrating RL and PK‐PD modeling was proposed to solve the multiobjective precision dosing problem of givinostat (Italfarmaco, ITF2357), a compound under clinical development for the treatment of polycythemia vera (PV) [33]. PV is a chronic life‐threatening neoplasm that is characterized by an overproduction of red blood cells, platelets (PLT), and white blood cells (WBC) [34, 35, 36, 37]. Givinostat inhibits the uncontrolled myeloproliferation of bone marrow cells in PV patients and the monitoring of platelets, thus decreasing the hematological parameter levels. Due to the high IIV of response to Givinostat and its small therapeutic index, Givinostat administration requires an adaptive dosing strategy in which PLT, WBC, and hematocrit (HCT) are monitored as both efficacy and toxicity endpoints. Here, RL was challenged to simultaneously optimize these three key hematological parameters, thus providing one of the first applications of model‐informed RL that copes with a multiobjective precision dosing problem.

The developed RL‐framework was first applied to derive a set of general adaptive dosing rules valid for all the PV patients (i.e., a single RL‐based controller for all the patients of the considered population [19, 28, 38]) and then to tailor the givinostat adaptive dosing protocol on each patient (i.e., a specific RL‐based controller for each patient [32]). For both the precision dosing scenarios, the obtained results were benchmarked against the clinical protocol proposed for the givinostat Phase III clinical trial [34]. To this regard, a further simulation exercise was conducted to evaluate the RL‐based treatment personalization as support of the drug development process to optimize the outcomes of the planned givinostat Phase III trial.

## 2 Methods

### 2.1 Givinostat Treatment of Polycythemia Vera

**FIGURE 1:**
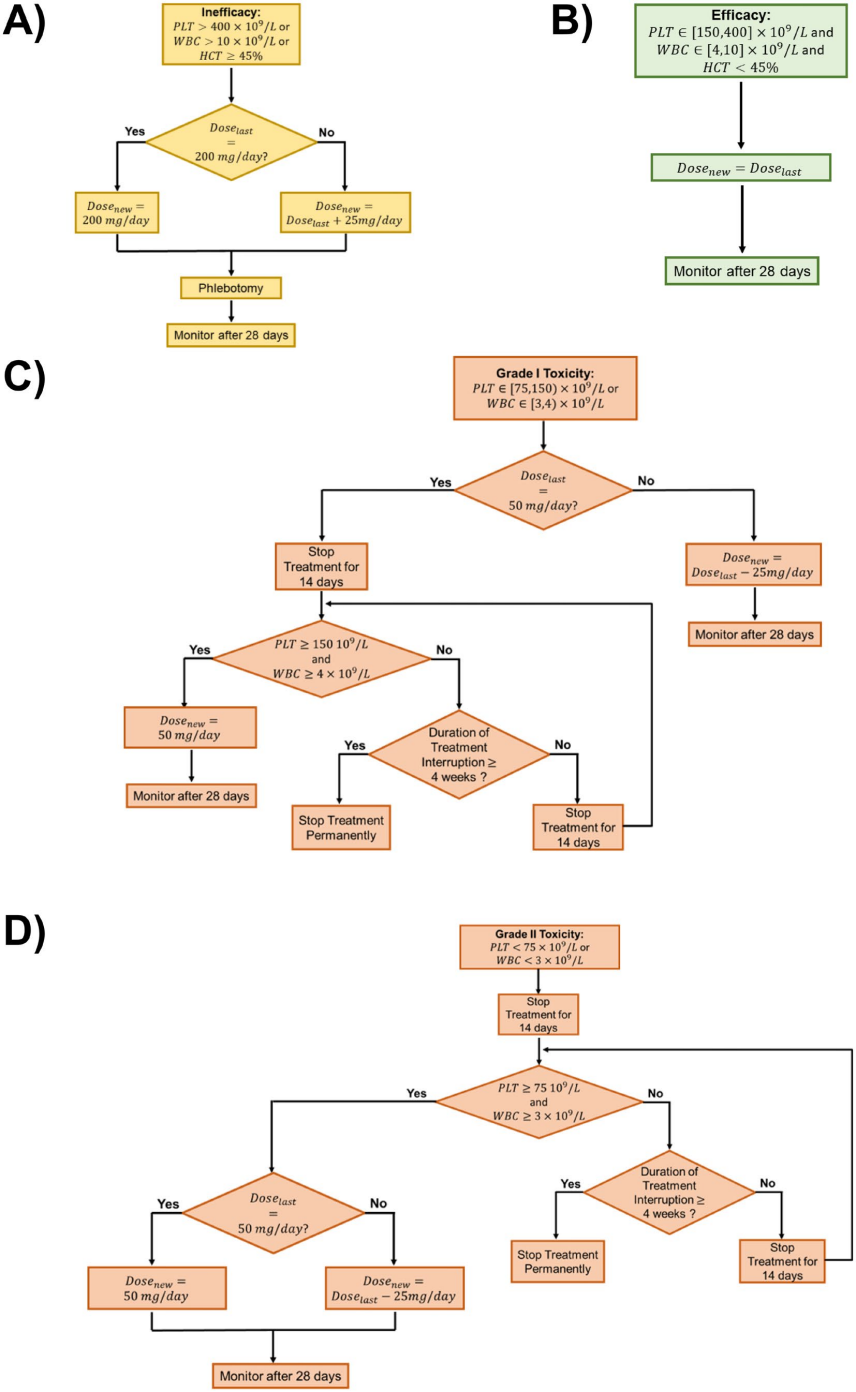
Givinostat adaptive‐dose protocol flow charts. Panel A illustrates the rules in case of inefficacy, Panel B in presence of efficacy. Panels C and D report the decisional steps made in presence of Grade I and Grade II toxicity, respectively. $\mathrm{Dos}e_{\mathrm{new}}$ = dose level for the next treatment cycle, $\mathrm{Dos}e_{\text{last}}$ = dose level for the previous treatment cycle, HCT = hematocrit, PLT = platelets, WBC = white blood cells.

The goal of givinostat treatment is to contrast the uncontrolled myeloproliferation in PV patients and, consequently, to simultaneously induce and maintain PLT, WBC, and HCT within an acceptable range (i.e., $\mathrm{PLT}\in \left[150,400\right]\times 10^{9}/L$, $\mathrm{WBC}\in \left[4,10\right]\times 10^{9}/L$, HCT<45%) [34]. These three hematological parameters are key endpoints for both efficacy and safety of givinostat treatment. Indeed, the treatment is ineffective if parameters are above the upper limits; instead, it induces some toxicity (i.e., thrombocytopenia and/or neutropenia) if they decrease below the lower limits. Toxicities can be of Grade 1 (i.e., moderate) if $\mathrm{PLT}\in \left[75,150\right)\times 10^{9}/L$ and/or $\mathrm{WBC}\in \left[3,4\right)\times 10^{9}/L$, or Grade 2 (i.e., severe) if $\;\mathrm{PLT}<75\times 10^{9}/L$ and/or $\mathrm{WBC}<3\times 10^{9}/L$. To maintain the hematological parameters within the efficacy range, a dose‐adaptive administration protocol based on the PLT, WBC, and HCT monitoring was proposed for the planned Phase III trial on givinostat [34]. In particular, givinostat can be administered at {50, 75, 100, 125, 150, 175, 200} mg/day dose level. For each patient, the treatment starts with a fixed dose of 100 mg/day. Only patients with a baseline HCT≥45% receive a preliminary phlebotomy to normalize this hematological parameter. Then, at the end of each 28‐day cycle the dose is adjusted (i.e., confirmed or increased/decreased by 25 mg/day) based on the measured PLT, WBC, and HCT values following rules schematized in Figure 1. In particular, the treatment is interrupted for 14 days (Figure 1, Panels C, D) if severe toxicities occur.

### 2.2 Setup of QL‐Framework for Givinostat Precision Dosing Problem

**FIGURE 2:**
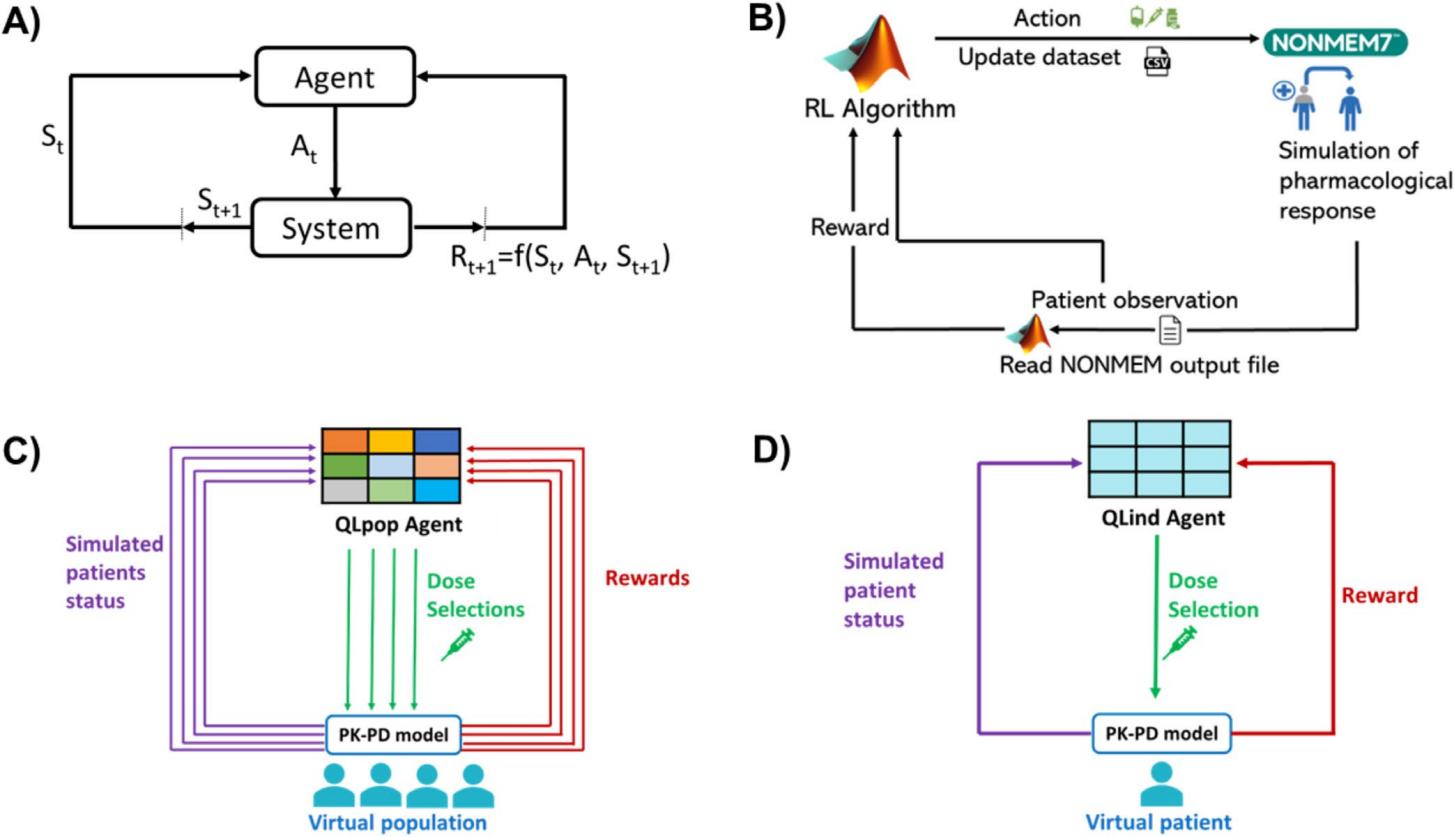
Panel A: Agent–System interplay in the MDP framework. Panel B: Integration of QL algorithm with PK‐PD simulation frameworks from an implementation point of view. Panel C: QL algorithm for the optimization of treatment in a population of patients (QLpop). Differently from Panel D, in this case the agent learns an optimal policy by considering the contributes of all patients in the population, rather than a single one. Panel D: QL‐based framework for treatment personalization (QLind). $A_{t}$ = selected agent action, PK‐PD = pharmacokinetics–pharmacodynamics, QL = Q‐Learning, RL = reinforcement learning, $R_{t+1}$ = reward value, $S_{t}$= current system state, $S_{t+1}$= next system state.

To optimize givinostat precision dosing problem with a Reinforcement Learning (RL) algorithm, it was necessary to formalize it as Markov Decision Process (MDP, Figure 2, Panel A) [18, 39]. A comprehensive theoretical description of MDP and RL is reported in Data S1. Therefore, the key modeler‐defined elements of an MDP, that is, system states ($S_{t}$), agent actions ($A_{t}$), and reward function ($R_{t}$), were designed in accordance with givinostat clinical setting. Briefly, system state (i.e., patient status) depends on the PLT, WBC, and HCT levels which are periodically monitored to guide dose adjustment (i.e., agent action). As the clinical goal is to achieve and maintain a Complete Hematological Response (CHR), which corresponds to simultaneously have $\mathrm{PLT}\in \left[150,400\right]\times 10^{9}/L$, $\mathrm{WBC}\in \left[4,10\right]\times 10^{9}/L$, and HCT<45% [34], dose adjustments are evaluated by a reward function accounting for all the three hematological parameters. Q‐Learning (QL) algorithm [18] was used to solve the givinostat MDP. A brief description of the method and its pseudocode are reported in Data S1. In the proposed framework, QL was trained by exploiting a simulation engine based on the available PK‐PD model for the givinostat effect on PLT, WBC, and HCT [34].

#### 2.2.1 Reward Function

The reward function was manually designed accordingly with the treatment goals of inducing and maintaining the three hematological parameters within their target ranges and of avoiding severe toxicities (i.e., $\mathrm{PLT}<75\times 10^{9}/L$ and/or $\mathrm{WBC}<3\times 10^{9}/L$). Therefore, in presence of severe myelosuppression, the reward function returns 0 (i.e., the lowest reward value); otherwise, it is given by the sum of three terms ($\text{Rewar}d_{\mathrm{PLT}},$ $\text{Rewar}d_{\mathrm{WBC}},\text{Rewar}d_{\mathrm{HCT}}$), one for each hematological parameter.

(1) $$\begin{array}{c} \text{Reward}=\left\{\begin{array}{c} 0\;\;\text{if}\;\mathrm{PL}T_{\mathrm{Obs}}<75\times 10^{9}/L\;\text{and}/\text{or}\;{\mathrm{WBC}}_{\mathrm{Obs}}<3\times 10^{9}/L\;\\ \begin{array}{cc} \text{Rewar}d_{\mathrm{PLT}}+\text{Rewar}d_{\mathrm{WBC}}+\text{Rewar}d_{\mathrm{HCT}} & \;\text{otherwise} \end{array}\; \end{array}\right. \end{array}$$

Each of the three terms in Equation (1) is given, in turn, by a weighted sum of three functions that take values in the range [0,1]:

(2) RewardPLT=β1·RewardPLT,Obs+β2·RewardPLT,Range+β3·RewardPLT,DerRewardWBC=β1·RewardWBC,Obs+β2·RewardWBC,Range+β3·RewardWBC,DerRewardHCT=β1·RewardHCT,Obs+β2·RewardHCT,Range+β3·RewardHCT,Derwithβ1=10,β2=7andβ3=5

The weights *β* are fixed, by design, to the values reported in Equation (2) for the three hematological parameters. In particular, the highest importance is given to the term $\text{Rewar}d_{\mathrm{Obs}}$ (Equations (3), (4), (5)) which scores the hematological parameter level (i.e., $\mathrm{PL}T_{\mathrm{Obs}}$, ${\mathrm{WBC}}_{\mathrm{Obs}}$, $\mathrm{HC}T_{\mathrm{Obs}})$ at the end of each treatment cycle with respect to the therapeutic window. This choice is aligned with the current clinical practice in which givinostat dose is adjusted according to the observed PLT, WBC, and HCT values at the end of treatment cycle (Figure 1). By definition, $\text{Rewar}d_{\mathrm{Obs}}$ gives a higher remuneration to actions (i.e., dose strategies) bringing the hematological parameter as close as possible to the middle of the normal range (Figures S1.7–S1.9).

(3) $$\begin{array}{c} \text{Rewar}d_{\mathrm{PLT},\mathrm{Obs}}\\ =\left\{\begin{array}{c} \begin{array}{cc} a\cdot e^{-0.01\cdot \left|\mathrm{PL}T_{\mathrm{obs}}-150\right|}+c & \text{if}\;\mathrm{PL}T_{\mathrm{Obs}}\in \left[75,150\right)\times 10^{9}/L\;\\ 0.5\cdot (1-e^{-0.06\cdot \left(\mathrm{PL}T_{\mathrm{Obs}}-150\right)}+0.5 & \text{if}\;\mathrm{PL}T_{\mathrm{Obs}}\in \left[150,275\right)\times 10^{9}/L\\ 0.5\cdot \left(1-e^{-0.06\cdot \left|\mathrm{PL}T_{\mathrm{Obs}}-150\right|}\right)+0.5 & \text{if}\;\mathrm{PL}T_{\mathrm{Obs}}\in \left[275,400\right)\times 10^{9}/L \end{array}\\ \begin{array}{cc} 0.5\cdot e^{-0.01\cdot \left(\mathrm{PL}T_{\mathrm{Obs}}-400\right)} & \text{if}\;\mathrm{PL}T_{\mathrm{Obs}}\geq 400\times 10^{9}/L\\ a=0.643 & c=-0.143 \end{array} \end{array}\right. \end{array}$$

(4) $$\begin{array}{c} \text{Rewar}d_{\mathrm{WBC},\mathrm{Obs}}\\ =\left\{\begin{array}{c} \begin{array}{cc} a\cdot e^{-0.01\cdot \left|37.5\cdot \mathrm{WB}C_{\mathrm{Obs}}-150\right|}+c & \text{if}\;\mathrm{WB}C_{\mathrm{Obs}}\in \left[3,4\right)\times 10^{9}/L\\ 0.5\cdot \left(1-e^{-0.06\cdot \left(37.5\cdot \mathrm{WB}C_{\mathrm{Obs}}-150\right)}\right)+0.5 & \text{if}\;\mathrm{WB}C_{\mathrm{Obs}}\in \left[4,7\right)\times 10^{9}/L\\ 0.5\cdot \left(1-e^{-0.06\cdot \left|40\cdot \mathrm{WB}C_{\mathrm{Obs}}-150\right|}\right)+0.5 & \text{if}\;\mathrm{WB}C_{\mathrm{Obs}}\in \left[7,10\right)\times 10^{9}/L \end{array}\\ \begin{array}{cc} 0.5\cdot e^{-0.01\cdot \left(40\cdot \mathrm{WB}C_{\mathrm{Obs}}-400\right)} & \text{if}\;\mathrm{WB}C_{\mathrm{Obs}}\geq 10\times 10^{9}/L\\ a=0.643 & c=-0.143 \end{array} \end{array}\right. \end{array}$$

(5) $$\begin{array}{c} \text{Rewar}d_{\mathrm{HCT},\mathrm{Obs}}\\ =\left\{\begin{array}{c} \begin{array}{cc} 0.5\cdot \left(1-e^{-0.06\cdot \left(8.89\cdot \mathrm{HC}T_{\mathrm{Obs}}-150\right)}\right)+0.5 & \text{if}\;\mathrm{HC}T_{\mathrm{Obs}}<22.5\%\\ 0.5\cdot \left(1-e^{-0.06\cdot \left|8.89\cdot \mathrm{HC}T_{\mathrm{Obs}}-400\right|}\right)+0.5\; & \text{if}\;\mathrm{HC}T_{\mathrm{Obs}}\in \left[22.5,45\right)\% \end{array}\\ \begin{array}{cc} 0.5\cdot e^{-0.01\cdot \left(8.89\cdot \mathrm{HC}T_{\mathrm{Obs}}-400\right)} & \text{if}\;\mathrm{HC}T_{\mathrm{Obs}}\geq 45\% \end{array} \end{array}\right. \end{array}$$

The second term, $\text{Rewar}d_{\text{Range}},$ weighted by $\beta_{2},$ considers the proportion of days in a treatment cycle in which the hematological parameter is within its target range (Equations (6), (7), (8)). This contribution captures the need of stably normalizing (i.e., avoiding oscillations within ineffective or toxic ranges) the hematological parameter as long as possible and not only at the end of the treatment cycle. Since this aspect of the treatment is relevant but less directly impactful on clinical decision‐making than end‐of‐cycle observations, $\beta_{2}$ was fixed lower than $\beta_{1}$.

(6) $$\begin{array}{c} \text{Rewar}d_{\mathrm{PLT},\text{Range}}=\left\{\begin{array}{c} \frac{\sum_{i}\mathrm{PL}T_{\text{cycle},i}}{\text{length}\left(\text{cycle}\right)}\\ \begin{array}{cc} \mathrm{PL}T_{\text{cycle},i}=1 & \text{if}\;\mathrm{PL}T_{\text{cycle},i}\in \left[150,400\right]\times 10^{9}/L\; \end{array}\\ \begin{array}{cc} \mathrm{PL}T_{\text{cycle},i}=0 & \text{if}\;\mathrm{PL}T_{\text{cycle},i}\notin \left[150,400\right]\times 10^{9}/L \end{array} \end{array}\right. \end{array}$$

(7) $$\text{Rewar}d_{\mathrm{WBC},\text{Range}}=\left\{\begin{array}{c} \frac{\sum_{i}{\mathrm{WBC}}_{\text{cycle},i}}{\text{length}\left(\text{cycle}\right)}\\ \begin{array}{cc} {\mathrm{WBC}}_{\text{cycle},i}=1 & \text{if}\;{\mathrm{WBC}}_{\text{cycle},i}\in \left[4,10\right]\times 10^{9}/L\; \end{array}\\ \begin{array}{cc} {\mathrm{WBC}}_{\text{cycle},i}=0 & \text{if}\;{\mathrm{WBC}}_{\text{cycle},i}\notin \left[4,10\right]\times 10^{9}/L \end{array} \end{array}\right.$$

(8) $$\text{Rewar}d_{\mathrm{HCT},\text{Range}}=\left\{\begin{array}{c} \frac{\sum_{i}{\mathrm{HCT}}_{\text{cycle},i}}{\text{length}\left(\text{cycle}\right)}\\ \begin{array}{cc} {\mathrm{HCT}}_{\text{cycle},i}=1 & \text{if}\;{\mathrm{HCT}}_{\text{cycle},i}<45\% \end{array}\\ \begin{array}{cc} {\mathrm{HCT}}_{\text{cycle},i}=0 & \text{if}\;{\mathrm{HCT}}_{\text{cycle},i}\geq 45\% \end{array} \end{array}\right.$$

The last term, $\text{Rewar}d_{\mathrm{Der}},$ weighted by $\beta_{3},$ is a function of the derivative, $\;y^{\prime }$, of the corresponding hematological parameters, where $y^{\prime }$ is calculated through the difference quotient over the two last values of a treatment cycle. Within the target range, this function (Equations (9), (10), (11) and Figures S1.4–S1.6) gives higher remuneration to stable trend (i.e., $y^{\prime }$ close to 0). Conversely, in presence of toxicity or inefficacy, it returns a higher reward if $y^{\prime }$ is positive or negative, respectively. In this way, also actions driving the hematological parameters to their target range are fostered.

(9) $$\text{Rewar}d_{\mathrm{PLT},\mathrm{Der}}\left(y_{\mathrm{PLT}}^{\prime }\right)=\left\{\begin{array}{c} \begin{array}{cc} \frac{0.2}{1+e^{-y^{\prime }}} & \text{if}\;y^{\prime }\leq 0\;\text{and}\;\mathrm{PL}T_{\mathrm{obs}}<150\times 10^{9}/L\\ \frac{1}{1+e^{-y^{\prime }}} & \text{if}\;y^{\prime }>0\;\text{and}\;\mathrm{PL}T_{\mathrm{Obs}}<150\times 10^{9}/L \end{array}\\ \begin{array}{cc} e^{\frac{y^{2}}{\psi }},\psi =1/\ln\left(5\right) & \text{if}\;\mathrm{PL}T_{\mathrm{Obs}}\in \left[150,400\right]\times 10^{9}/L \end{array}\\ \begin{array}{cc} \frac{1}{1+e^{-|y^{\prime }|}} & \text{if}\;y^{\prime }<0\;\text{and}\;\mathrm{PL}T_{\mathrm{Obs}}>400\times 10^{9}/L\;\\ \frac{0.2}{1+e^{-y^{\prime }}} & \text{if}\;y^{\prime }\geq 0\;\text{and}\;\mathrm{PL}T_{\mathrm{Obs}}>400\times 10^{9}/L \end{array} \end{array}\right.$$

(10) $$\text{Rewar}d_{\mathrm{WBC},\mathrm{Der}}\left(y_{\mathrm{WBC}}^{\prime }\right)=\left\{\begin{array}{c} \begin{array}{cc} \frac{0.2}{1+e^{-y^{\prime }}} & \text{if}\;y^{\prime }\leq 0\;\text{and}\;{\mathrm{WBC}}_{\mathrm{obs}}<4\times 10^{9}/L\\ \frac{1}{1+e^{-y^{\prime }}} & \text{if}\;y^{\prime }>0\;\text{and}\;{\mathrm{WBC}}_{\mathrm{Obs}}<4\times 10^{9}/L \end{array}\\ \begin{array}{cc} e^{\frac{y^{2}}{\psi }},\psi =1/\ln\left(5\right) & \text{if}\;{\mathrm{WBC}}_{\mathrm{Obs}}\in \left[4,10\right]\times 10^{9}/L \end{array}\\ \begin{array}{cc} \frac{1}{1+e^{-|y^{\prime }|}} & \text{if}\;y^{\prime }<0\;\text{and}\;{\mathrm{WBC}}_{\mathrm{Obs}}>10\times 10^{9}/L\;\\ \frac{0.2}{1+e^{-y^{\prime }}} & \text{if}\;y^{\prime }\geq 0\;\text{and}\;{\mathrm{WBC}}_{\mathrm{Obs}}>10\times 10^{9}/L \end{array} \end{array}\right.$$

(11) $$\text{Rewar}d_{\mathrm{HCT},\mathrm{Der}}\left(y_{\mathrm{HCT}}^{\prime }\right)=\left\{\begin{array}{c} \begin{array}{cc} e^{\frac{y^{2}}{\psi }},\psi =1/\ln\left(5\right) & \text{if}\;\;{\mathrm{HCT}}_{\mathrm{Obs}}<45\% \end{array}\\ \begin{array}{cc} \frac{1}{1+e^{-|y^{\prime }|}} & \text{if}\;y^{\prime }<0\;\text{and}\;{\mathrm{HCT}}_{\mathrm{Obs}}\geq 45\% \end{array}\\ \begin{array}{cc} \frac{0.2}{1+e^{-y^{\prime }}} & \text{if}\;y^{\prime }\geq 0\;\text{and}\;{\mathrm{HCT}}_{\mathrm{Obs}}\geq 45\% \end{array} \end{array}\right.$$

#### 2.2.2 System States

The system (i.e., patient) state is described by a tuple of four elements, $X=\left\{\mathrm{PL}T_{\text{Discr}},\mathrm{WB}C_{\text{Discr}},\mathrm{HC}T_{\text{Discr}},\text{PrevDose}\right\}.$ $\mathrm{PL}T_{\text{Discr}.},\mathrm{WB}C_{\text{Discr}.},\mathrm{HC}T_{\text{Discr}.}$ (Equations (12), (13), (14)) are the observations of PLT, WBC, and HCT at the monitored day ($\mathrm{PL}T_{\mathrm{Obs}},\mathrm{WB}C_{\mathrm{Obs}},\mathrm{HC}T_{\mathrm{Obs}})$ categorized according to the inefficacy, efficacy, and toxicity definition suggested by the clinicohematological European Leukemia Network criteria [40].

(12) $$\begin{array}{c} \mathrm{PL}T_{\text{Discr}}\left(\mathrm{PL}T_{\mathrm{Obs}}\right)\\ =\left\{\begin{array}{c} \begin{array}{cc} 1 & \text{if}\;\mathrm{PL}T_{\mathrm{Obs}}<75\times 10^{9}/L\;\left(\text{Severe Thrombocytopenia}\right)\\ 2 & \text{if}\;\mathrm{PL}T_{\mathrm{Obs}}\in \left[75,150\right)\times 10^{9}/L\;\left(\text{Moderate Thrombocytopenia}\right) \end{array}\\ \begin{array}{cc} 3 & \text{if}\;\mathrm{PL}T_{\mathrm{Obs}}\in \left[150,400\right]\times 10^{9}/L\;\left(\text{Efficacy}\right)\\ 4 & \text{if}\;\mathrm{PL}T_{\mathrm{Obs}}>400\times 10^{9}/L\;\left(\text{Unefficacy}\right) \end{array}\; \end{array}\right. \end{array}$$

(13) $$\begin{array}{c} {\mathrm{WBC}}_{\text{Discr}}\left({\mathrm{WBC}}_{\mathrm{Obs}}\right)\\ =\left\{\begin{array}{c} \begin{array}{cc} 1 & \text{if}\;{\mathrm{WBC}}_{\mathrm{Obs}}<3\times 10^{9}/L\;\left(\text{Severe Neutropenia}\right)\\ 2 & \text{if}\;{\mathrm{WBC}}_{\mathrm{Obs}}\in \left[3,4\right)\times 10^{9}/L\;\left(\text{Moderate Neutropenia}\right) \end{array}\\ \begin{array}{cc} 3 & \text{if}\;{\mathrm{WBC}}_{\mathrm{Obs}}\in \left[4,10\right]\times 10^{9}/L\;\left(\text{Efficacy}\right)\\ 4 & \text{if}\;{\mathrm{WBC}}_{\mathrm{Obs}}>10\times 10^{9}/L\;\left(\text{Unefficacy}\right) \end{array}\; \end{array}\right. \end{array}$$

(14) $$\mathrm{HC}T_{\text{Discr}}\left(\mathrm{HC}T_{\mathrm{Obs}}\right)=\left\{\begin{array}{c} \begin{array}{cc} 1 & \text{if}\;\mathrm{HC}T_{\mathrm{Obs}}<45\%\left(\text{Efficacy}\right) \end{array}\;\\ \begin{array}{cc} 2 & \text{if}\;\mathrm{HC}T_{\mathrm{Obs}}\geq 45\%\left(\text{Unefficacy}\right) \end{array} \end{array}\right.$$

PrevDose contains the information on the last administered givinostat dose. Possible values of PrevDose are the clinically available dose levels, that is, {50, 75, 100, 125, 150, 175, 200} mg/day, combined with a sign that is negative if the treatment is interrupted due to severe toxicity and positive otherwise (Equation 15). Instead, PrevDose=0 codes the treatment interruption due to moderate toxicities ($\mathrm{PLT}\in \left[75,150\right)\times 10^{9}/L\;\text{and}/\text{or}\;\mathrm{WBC}\in \left[3,4\right)\times 10^{9}/L$ and dose triggering interruption of 50 mg/day, Figure 1, Panel C). This coding strategy allows us to compactly store all the information about temporary interruption (i.e., triggering dose and toxicity severity) that is necessary to correctly select the resumption dose (Figure 1, Panels C,D). Furthermore, PrevDose=−1 was a flag for the treatment start, when the initial dose has to be selected by the agent.

(15) $$\begin{array}{c} \text{PrevDose}\\ =\left\{\begin{array}{c} \begin{array}{cc} -1 & \left(\text{Initial State}\right)\\ -50,-75,-100,-125,-150,-175,-200 & \left(\text{Treatment Interruption}\;\mathrm{due}\;\text{to severe}\;\mathrm{tox}.\right) \end{array}\\ \begin{array}{cc} 0 & \left(\text{Treatment Interruption}\;\mathrm{due}\;\text{to moderate}\;\mathrm{tox}.\right)\\ 50,75,100,125,150,175,200 & \begin{array}{cc} & \left(\text{Treatment not Interrupted}\right) \end{array} \end{array} \end{array}\right. \end{array}$$

Combining all the values of $\mathrm{PL}T_{\text{Discr}.},\mathrm{WB}C_{\text{Discr}.},\mathrm{HC}T_{\text{Discr}.}$, and each PrevDose≠−1, 480 states were defined. Then, combining PrevDose=−1 with all $\mathrm{PL}T_{\text{Discr}.}\geq 3,\mathrm{WBC}\geq 3,\mathrm{HCT}\geq 1$, 8 initial states were added. However, as all PV patients have at least one hematological parameter above the target range at the baseline [34], the tuple $\left\{\mathrm{PL}T_{\text{Discr}.}=3,\mathrm{WB}C_{\text{Discr}.}=3,\mathrm{HC}T_{\text{Discr}.}=1,\text{PrevDose}=-1\right\}$ was discarded, obtaining 487 states.

#### 2.2.3 Agent Actions

The actions available to the QL‐agents consist in selecting the dose for each treatment cycle, which lasts 28 days accordingly to the clinical protocol. Only in presence of temporary interruption its duration is reduced to 14 days (Panels C and D of Figure 1). Actions were designed accounting for some safety constraints, coherently with the clinical protocol. Gradual dose changes (i.e., ±1 level with respect to the current amount) and safety treatment interruption criteria were imposed. However, the agent has a higher degree of freedom in making decisions with respect to the clinical rules (Figure 1), to explore and identify potentially better personalized treatments. As schematized in Table 1, agent was allowed to select alternative treatment starting doses (i.e., the dose for the first cycle of the therapy) with respect to the standard one (i.e., 100 mg/day). Further, in absence of severe toxicity (i.e., efficacy or inefficacy), agent was not forced to maintain the current dose level (i.e., D=) but could perform stepwise changes (i.e., increase/decrease by one level, D+/D−). Finally, a higher flexibility was given to the selection of the treatment resumption dose after the temporary interruption due to severe toxicity events.

**TABLE 1:** Possible agent actions stratified by system/patient state.

PrevDose	QL‐agent actions
Initial state	
−1	50,75,100,125,150,175,200[mg/day]
States without severe toxicities: $\mathrm{PL}T_{\text{Discr}.}\geq 3,\mathrm{WB}C_{\text{Discr}.}\geq 3,\mathrm{HC}T_{\text{Discr}.}\geq 1$	
50	D=,D+
75	D=,D+,D−
100	D=,D+,D−
125	D=,D+,D−
150	D=,D+,D−
175	D=,D+,D−
200	D=,D−
States with moderate/grade 1 toxicity: $\mathrm{PL}T_{\text{Discr}.}=2$ and/or $\mathrm{WB}C_{\text{Discr}.}=2,\mathrm{HC}T_{\text{Discr}.}\geq 1$	
50	0 mg/day for 14 days (temporary treatment interruption)^a^
75	D=,D+,D−
100	D=,D+,D−
125	D=,D+,D−
150	D=,D+,D−
175	D=,D+,D−
200	D=,D−
States in which treatment can be resumed following temporary interruption due to moderate/grade 2 toxicity: $\mathrm{PL}T_{\text{Discr}.}=3$ and $\mathrm{WB}C_{\text{Discr}.}=3,\mathrm{HC}T_{\text{Discr}.}\geq 1$	
0	0, 50 [mg/day]
States with Severe/Grade 2 Toxicity: $\mathrm{PL}T_{\text{Discr}.}=1$ and/or $\mathrm{WB}C_{\text{Discr}.}=1,\mathrm{HC}T_{\text{Discr}.}\geq 1$	
∀PrevDose	0 mg/day for 14 days (temporary treatment interruption)^a^
States in which treatment can be resumed following temporary interruption due to severe/grade 2 toxicity: $\mathrm{PL}T_{\text{Discr}.}=2$ and $\mathrm{WB}C_{\text{Discr}.}=2,\mathrm{HC}T_{\text{Discr}.}\geq 1$	
−50	0, 50 [mg/day]
−75	0, 50, 75 [mg/day]
−100	0, 50, 75, 100 [mg/day]
−125	0, 50, 75, 100, 125 [mg/day]
−150	0, 50, 75, 100, 125, 150 [mg/day]
−175	0, 50, 75, 100, 125, 150, 175 [mg/day]
−200	0, 50, 75, 100, 125, 150, 175, 200 [mg/day]

Abbreviations: $\mathrm{HC}T_{\text{Discr}.}$ = discretized hematocrit level, PrevDose = last administered givinostat dose, QL = Q‐learning, $\mathrm{WB}C_{\text{Discr}.}$ = discretized white blood cell level, $\mathrm{PL}T_{\text{Discr}.}$ = discretized platelet level.

^a^ Safety constraint of givinostat phase III clinical protocol.

### 2.3 Implementation of the Framework

A simulation engine based on the available givinostat PK‐PD model [34] was embedded in the QL‐framework to predict the outcome and the reward of each dosing strategy for each patient (model details in Data S2). Residual Unexplained Variability (RUV) was not considered in simulations, assuming the status evolution of the PV patient was fully described by its PK‐PD model. NONMEM version 7.3.0 (ICON plc) was used to run simulations of the givinostat PK‐PD model for each dosing strategy. MATLAB was used to develop the decisional algorithm, represented by the QL‐agents, exploiting the already available simulation platform [41], (Figure 2, Panel B).

### 2.4 Learning a Unique Adaptive Dosing Protocol for the Whole Population With QL

**FIGURE 3:**
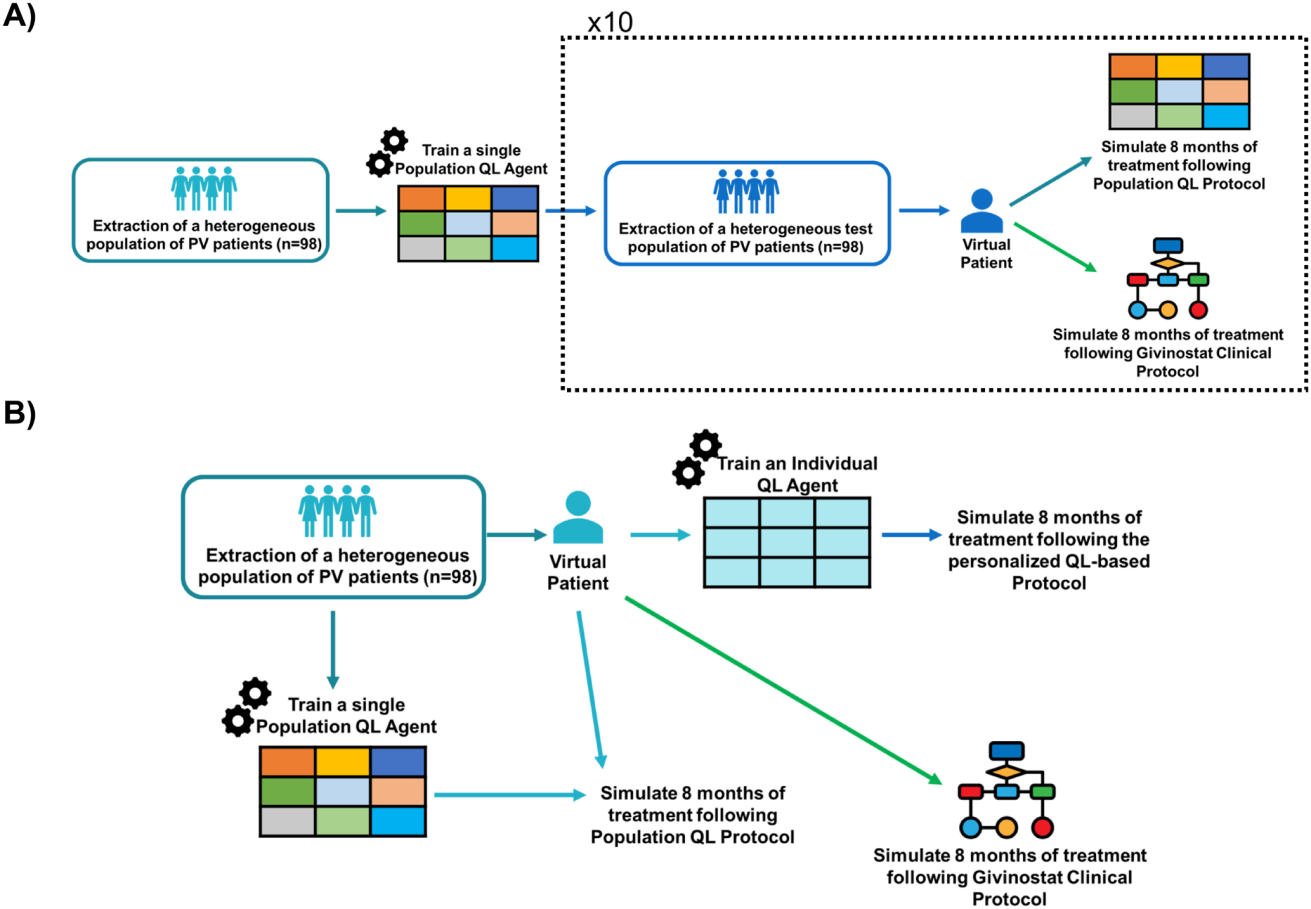
Evaluation of the two QL‐based precision dosing strategies. Panel A illustrates the implemented framework to assess the performances of a general QL protocol (QL_pop_). Its robustness against interindividual variability was compared with givinostat Phase III protocol on 10 external test sets. Panel B shows the evaluation framework of personal QL‐agents (QL_ind_). In this case, the comparison was performed against the clinical and the QL_pop_‐agent protocols. PV = Polycythemia Vera, QL = Q‐Learning.

The developed QL‐framework was first applied to derive a set of adaptive dosing rules for givinostat suitable for all the PV patients. To this end, a unique QL‐agent (QL_pop_‐agent) was trained on a heterogeneous population of virtual PV patients [19, 28, 38] and the optimal policy was learned by gathering the rewards from all the individuals (Figure 2, Panel C). A treatment duration of 8 months, that is, the average time to achieve a stable hematological response [34], was considered. Then, the QL_pop_‐agent performances were evaluated on 10 test sets of virtual individuals and benchmarked on the same pools of virtual patients by the clinical protocol proposed for a Phase III study (Figure 3, Panel A). Both training and test sets were composed of 98 virtual patients generated through a stratified random sampling strategy (details in Data S3) to obtain heterogeneous virtual populations in terms of treatment response and baseline conditions. Individual parameters and covariates of virtual patients were extracted from the parameter distributions of the givinostat population PK/PD model and the covariate distributions of subjects on which the model was originally estimated (see Data S2 and S3).

### 2.5 Learning Patient‐Specific Adaptive Dosing Strategies With QL

QL‐framework was also used to individually tailor the adaptive dosing rules of givinostat for each single patient. To this aim, an individual‐oriented QL‐approach based on a set of personal QL‐agents (QL_ind_‐agents, Figure 2, Panel D) [32] was adopted. For each patient, a personal QL_ind_‐agent was trained over an 8‐month period, and an optimal personalized dosing policy was derived. The performances of the QL_ind_‐agents were assessed on a population of 98 virtual patients with high heterogeneity in terms of treatment response and baseline conditions (details on its generation in Data S3). Givinostat clinical protocol was used as a benchmark in the simulation‐based framework reported in Panel B of Figure 3, where each virtual patient is treated with both adaptive dosing strategies. Furthermore, to measure the potentialities of this deeper treatment personalization, QL_ind_‐agents were also compared with the QL_pop_ approach on the same pool of virtual individuals. The comparison of QL_pop_ and QL_ind_‐agents was intentionally performed on the patient population on which the QL_pop_‐agent was trained (Figure 3, Panel B) to consider its most favorable conditions.

## 3 Results

### 3.1 Unique QL‐Based Adaptive Dosing Protocol for the Whole Population

The QL_pop_‐agent was trained on a virtual population of 98 PV patients (hyperparameters fixed for the training in Data S4) with high heterogeneity in treatment response to learn a dosing protocol that is suitable for as large a number of subjects as possible. As detailed in Data S4, the learned policy achieved lower CHR rates, even in the training population. Indeed, as severe myelosuppression events were strongly penalized in the reward function (reward = 0 as reported in Equation 1), the QL_pop_ dosing policy was biased to prefer lower doses which are unlikely to provoke severe toxicities but also to enable normalization of the three hematological parameters (Figures S4.1 and S4.2). Therefore, to increase the efficacy rate of the QL‐based general protocol, the previous definition of the reward function was revised. In particular, the too strong penalty for severe toxicities was smoothed by replacing Equation (1) with Equation (16), and consequently, Equations (3 and 4) with Equations (17) and (18) (Figures S1.1 and S1.2).

(16) $$\text{Reward}=\text{Rewar}d_{\mathrm{PLT}}+\text{Rewar}d_{\mathrm{WBC}}+\text{Rewar}d_{\mathrm{HCT}}$$

(17) $$\begin{array}{c} \text{Rewar}d_{\mathrm{PLT},\mathrm{Obs}}\\ =\left\{\begin{array}{c} \begin{array}{cc} a\cdot e^{-0.01\cdot \left|\mathrm{PL}T_{\mathrm{obs}}-150\right|}+c & \text{if}\;\mathrm{PL}T_{\mathrm{Obs}}<150\times 10^{9}/L\\ 0.5\cdot (1-e^{-0.06\cdot \left(\mathrm{PL}T_{\mathrm{Obs}}-150\right)}+0.5 & \text{if}\;\mathrm{PL}T_{\mathrm{Obs}}\in \left[150,275\right)\times 10^{9}/L \end{array}\\ \begin{array}{cc} 0.5\cdot \left(1-e^{-0.06\cdot \left|\mathrm{PL}T_{\mathrm{Obs}}-150\right|}\right)+0.5 & \text{if}\;\mathrm{PL}T_{\mathrm{Obs}}\in \left[275,400\right)\times 10^{9}/L\\ 0.5\cdot e^{-0.01\cdot \left(\mathrm{PL}T_{\mathrm{Obs}}-400\right)} & \text{if}\;\mathrm{PL}T_{\mathrm{Obs}}\geq 400\times 10^{9}/L \end{array}\\ \begin{array}{cc} a=0.643 & c=-0.143 \end{array} \end{array}\right. \end{array}$$

(18) $$\begin{array}{c} \text{Rewar}d_{\mathrm{WBC},\mathrm{Obs}}\\ =\left\{\begin{array}{c} \begin{array}{cc} a\cdot e^{-0.01\cdot \left|37.5\cdot \mathrm{WB}C_{\mathrm{Obs}}-150\right|}+c & \text{if}\;\mathrm{WB}C_{\mathrm{Obs}}<4\times 10^{9}/L\\ 0.5\cdot \left(1-e^{-0.06\cdot \left(37.5\cdot \mathrm{WB}C_{\mathrm{Obs}}-150\right)}\right)+0.5 & \text{if}\;\mathrm{WB}C_{\mathrm{Obs}}\in \left[4,7\right)\times 10^{9}/L\; \end{array}\\ \begin{array}{cc} 0.5\cdot \left(1-e^{-0.06\cdot \left|40\cdot \mathrm{WB}C_{\mathrm{Obs}}-150\right|}\right)+0.5 & \text{if}\;\mathrm{WB}C_{\mathrm{Obs}}\in \left[7,10\right)\times 10^{9}/L\\ 0.5\cdot e^{-0.01\cdot \left(40\cdot \mathrm{WB}C_{\mathrm{Obs}}-400\right)} & \text{if}\;\mathrm{WB}C_{\mathrm{Obs}}\geq 10\times 10^{9}/L \end{array}\\ \begin{array}{cc} a=0.643 & c=-0.143 \end{array} \end{array}\right. \end{array}$$

**FIGURE 4:**
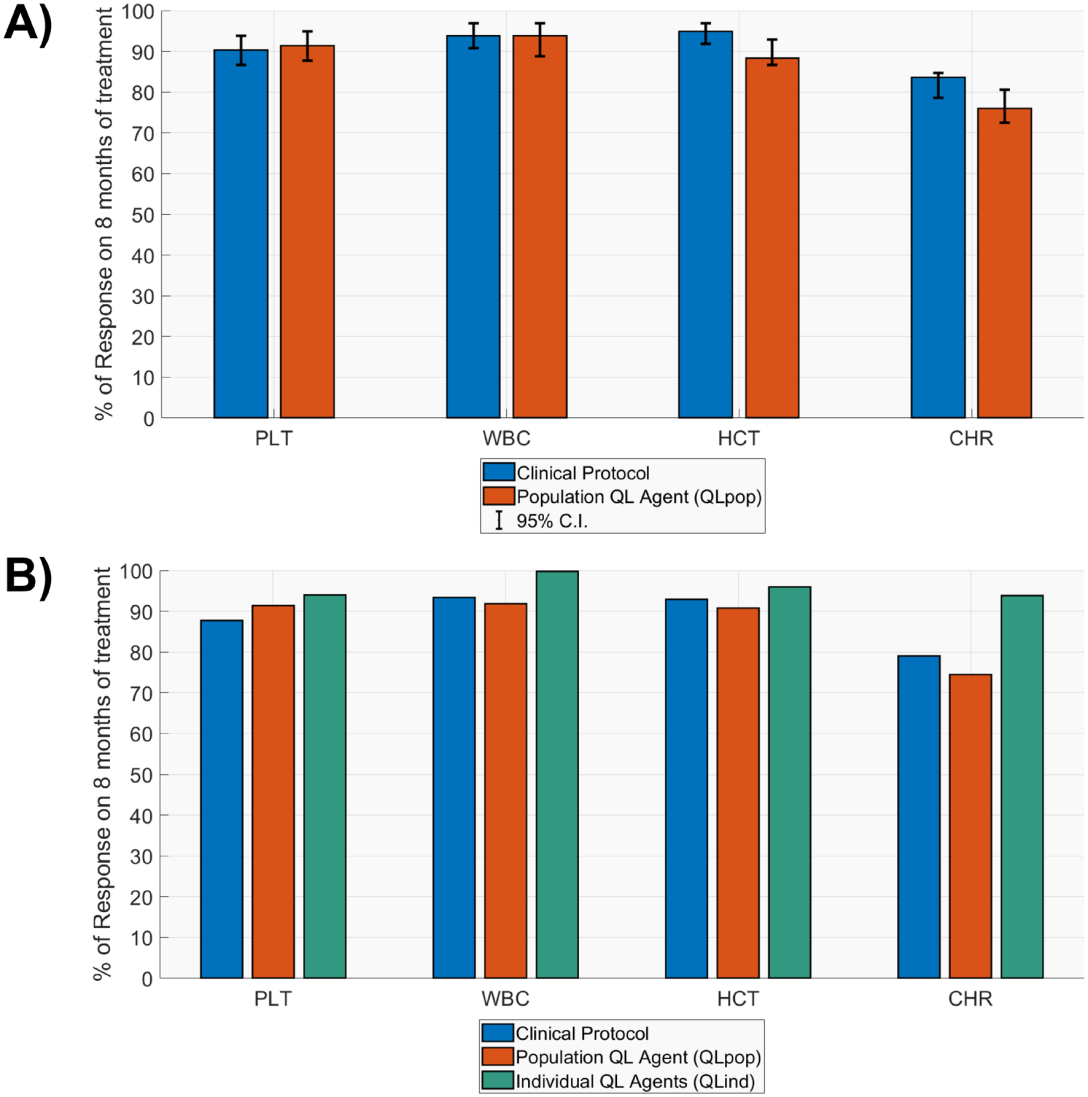
Summary of efficacy outcomes for the preliminary evaluation of Givinostat precision dosing problem (Panel A) and the multiobjective treatment optimization with personal QL‐agents (QL_ind_‐agents, Panel B). CHR = complete hematological response, HCT = hematocrit, PLT = platelets, QL = Q‐Learning, WBC = white blood cells.

As shown in Data S4, with the new reward function, the CHR rate achieved by the QL_pop_‐agent on the training population significantly increased. Then, its performance was further evaluated on 10 different test sets and benchmarked against the results obtained by the phase III protocol on the same 10 virtual populations. After 8 months of treatment, the Q_pop_‐agent and the clinical rules induced similar rates of both single hematological parameter responses and CHR (Figure 4, Panel A). The time necessary to achieve a stable CHR was comparable for the two protocols (Table 2), too.

**TABLE 2:** Comparison between QL_pop_‐agent and the clinical protocol proposed for the Givinostat Phase III study on 10 test sets composed of 98 virtual patients.

QL_pop_‐agent	Clinical protocol
Days until stable CHR [95% C.I.]	
116	110
[35, 211]	[41, 214]
% of patients with at least one severe toxicity event [95% CI]^a^	
40.30	14.28
[37.75, 44.89]	[11.22, 17.34]
% of Days on 8‐month treatment with severe toxicity	
17.33 [3.63, 46.77]	14.67 [5.5, 29.11]
% of Days on 8‐month treatment with $\mathrm{PLT}\in \left[150,400\right]\times 10^{9}/L$	
68.44 [24.34, 100]	76.44 [32.23, 100]
% of Days on 8‐month treatment with $\mathrm{WBC}\in \left[4,10\right]\times 10^{9}/L$	
82.22 [38.57, 100]	80.67 [37.80, 100]
% of Days on 8‐month treatment with HCT < 45%	
92.22 [22.14, 100]	90.55 [44.38, 100]
% of Days on 8‐month treatment with CHR	
45.33 [4.18, 83.62]	52.55 [9.70, 80.80]

*Note:* Median and 95% CI were computed for each metric within each test set. Here, the median of the 10 median values, the 2.5° percentile of the 10 2.5° percentiles, and the 97.5° percentile of the 10 97.5° percentiles are reported.

Abbreviations: CI = confidence interval, CHR = complete hematological response, HCT = hematocrit, PLT = platelets, QL = Q‐Learning, WBC = white blood cells.

^a^ 95% CI computed only across the 10 test sets, as for each of them a single value is obtained.

Conversely, QL_pop_‐agent showed lower safety performances with respect to the clinical protocols as a higher percentage of severe toxicity events was observed in the 10 test sets. Such episodes mainly occurred at the beginning of treatment (Figure S4.1, Panel B) as QL_pop_‐agent adopted higher starting doses (200 mg or 150 mg depending on whether $\mathrm{WBC}>10\times 10^{9}/L$ or $\mathrm{WBC}\in \left[4,10\right]\times 10^{9}/L$) compared to the 100 mg of the clinical protocol. Interestingly, QL_pop_‐agent estimated the same dosing rules of the clinical protocol for all patient efficacy status (i.e., maintaining the current dose level, Figure 1, Panel B).

### 3.2 QL‐Based Patient‐Specific Adaptive Dosing Protocols

The individual‐oriented QL‐approach was applied to the same 98‐patient virtual population used to train the QL_pop_‐agent (Figure 3, Panel B). For each patient, a personal QL_ind_‐agent, characterized by the reward function described in Equations ((1), (2), (3), (4), (5), (6), (7), (8), (9), (10), (11)), was trained to specifically optimize the givinostat treatment over an 8‐month period in the given subject (hyperparameters fixed for the training procedure in Data S4). Then, on the same population, the performances of the QL_ind_‐agents were compared with those of the clinical protocol and of the previously developed QL_pop_‐agent by leveraging the simulation‐based framework in Panel B of Figure 3. As shown in Figure 4, Panel B, and Table 3, the QL_ind_‐agents outperformed both the clinical and QL_pop_‐based protocols. Indeed, after 8 months of treatment, the QL_ind_‐based protocols achieved the highest rates for the single hematological parameter responses as well as for CHR (93% vs. 79% and 75%). In addition, considering the whole treatment period, the QL_ind_‐based protocols were able to simultaneously maintain PLT, WBC, and HCT in the corresponding target ranges for a higher percentage of days. QL_ind_‐agents dramatically reduced the time needed to reach a stable CHR with respect to clinical and QL_pop_‐based rules (i.e., 47 days vs. 105 and 99 days, respectively). More in detail, from day 84, QL_ind_‐based dosing strategies were able to maintain a CHR in over 90% of the patients (Figure S5.5). This result is mainly due to the ability of QL_ind_‐agents to correctly select the highest/lowest dose (Figures S5.1 and S5.3) from the first cycle or to detect patients tolerating a loading dose (Figure S5.2). Once the optimal dose was identified, QL_ind_‐agents were able to stabilize all the hematological parameters with few dose adjustments (i.e., median of 1, Panel A of Figure S5.8). As regards treatment safety, the individual QL‐approach outperforms the two benchmarks by totally avoiding severe toxicities and reducing the moderate episodes (Table 3 and Figures S5.4, S5.6, and S5.7), thus minimizing also the treatment discontinuation rates (Figure S5.8). The customization of the initial dose was central for QL_ind_‐agents in enhancing the safety outcomes of givinostat treatment. Indeed, both the fixed starting dose rules of the QL_pop_‐agent and the clinical protocol showed higher toxicity rates (Figures S5.6 and S5.7) at the beginning of treatment and, consequently, higher treatment interruptions (Figure S5.8, Panels B,C).

**TABLE 3:** Comparison between the performances of QL_ind_‐agent, QL_pop_‐agent, and clinical protocol on the same virtual population of 98 PV patients.

QL_pop_‐agent	QL_ind_‐agents	Clinical protocol
Days until stable CHR [95% CI]		
99 [35, 211]	47 [25, 147]	105 [41, 214]
% of patients with at least one severe toxicity event		
43.88	0.00	12.24
% of Days on 8‐month treatment with severe toxicity		
13.78 [2.03, 54.25]	0 [0, 0]	17.78 [10.67, 28.44]
% of Days on 8‐month treatment with $\mathrm{PLT}\in \left[150,400\right]\times 10^{9}/L$		
71.78 [24.87, 100]	91.56 [41.67, 100]	79.78 [28.62, 100]
% of Days on 8‐month treatment with $\mathrm{WBC}\in \left[4,10\right]\times 10^{9}/L$		
85.33 [20.11, 100]	88.44 [64.8, 100]	81.33 [34.98, 100]
% of Days on 8‐month treatment with HCT < 45%		
96.22 [20.11, 100]	94.00 [46.13, 100]	92.44 [44.38, 100]
% of Days on 8‐month treatment with CHR		
50.89 [7.51, 88.00]	75.56 [33.44, 88.89]	53.33 [11.82, 81.38]

*Note:* For each metric, the median and 95% CI in the population are reported.

Abbreviations: CI = confidence interval, CHR = complete hematological response, HCT = hematocrit, PLT = platelets, QL = Q‐Learning, WBC = white blood cells.

## 4 Discussion

Recently, the interest in AI/ML has rapidly spread among pharmacometricians, as witnessed by the increasing number of AI/ML applications for several pharmacometric tasks [13, 14, 42]. Among them, the integration of RL with PK/PD models for precision dosing purposes raised special attention [7, 16, 43]. In most applications of this hybrid framework, a unique RL‐agent was trained to derive a general dose‐adaptive protocol that optimizes pharmacological treatment in a population of patients [19, 28, 38]. Recently, a novel individual‐oriented framework coupling personal RL‐agents with patient digital twins (i.e., patient‐specific PK‐PD models) was proposed in order to customize adaptive‐dose strategies for each individual patient [32].

Here, a hybrid framework integrating the QL‐algorithm and PK‐PD modeling (Figure 2) was applied to solve the precision dosing problem relating to PV treatment with givinostat. It represents an interesting and not trivial clinical example of a dose‐adaptive regimen (Figure 1) for which dose adjustments depend on multiple key parameters (i.e., PLT, WBC, and HCT) that are simultaneously considered as efficacy and safety endpoints. Although dose adaptation based on multiple endpoints represents a common clinical practice, especially in the oncology field, RL applications to multiobjective optimization problems are still extremely limited [43]. In addition, the high IIV affecting the hematological parameters dynamics and the givinostat response [34] further challenges the identification of a treatment regimen that can be adequate for all PV patients. Indeed, dosing rules effective for some patients could not be optimal for other individuals exhibiting atypical behaviors. This suggested that the givinostat treatment could potentially benefit from a customization of the dose adjustment rules at the individual patient level.

Before applying QL, the sequential decision‐making process relating to givinostat‐based treatment has to be formalized within the MDP domain. This means providing an appropriate configuration for the modeler‐defined components of the MDP (i.e., states, actions, and reward function) [17]. To this end, system/patient states were defined, including all the relevant information to inform the action choice (i.e., dose selection). Previously administered dose and a categorization of the three biomarker levels (Equations (12), (13), (14)) were encoded in the state definition. This formalization allowed the QL‐agent to identify 487 distinct clinical conditions in which givinostat dose can be adjusted, resulting in more specific and complex adaptive dosing rules than the clinical protocol, which applied a coarser stratification of situations in which to take dosing decisions. QL‐agent actions were defined to allow the exploration of different dosing strategies potentially better than the clinical protocol and, simultaneously, to obtain clinically acceptable rules. To this regard, only clinically available dose levels were considered, and some safety constraints (i.e., gradual dose variation, interruption due to severe toxicities) were imposed on the QL‐agents. Differently from the clinical protocol, QL‐agents had more degrees of freedom in the selection of both initial and resumption doses, as well as in dose adjustments in the absence of severe toxicity. Finally, the goal of givinostat treatment was formalized in the reward function described by Equations ((1), (2), (3), (4), (5), (6), (7), (8), (9), (10), (11)), which were manually designed by leveraging the literature available clinical knowledge [33, 34]. More in detail, due to the multiobjective optimization problems, three equally weighted terms, one for each hematological parameter, were introduced to enable their simultaneous optimization (Equation 1). Each of the three terms defined for the single hematological parameter was, in turn, the result of a weighted sum in which the highest importance was given to the observations of PLT, WBC, and HCT at the end of the treatment cycle, as they drive clinical dose adjustments (Figure 1).

Once the QL‐framework has been appropriately defined, it has to be integrated with a PK/PD model to simulate patient responses to the agent's dosing actions. The choice of the model to use as the simulation engine is of paramount importance, as the success of the RL approach intrinsically depends on the reliability of the model predictions [17]. In the case of givinostat, a PK/PD model describing the hematological response in PV patients was used [34]. This model had been previously developed and robustly validated based on clinical data, demonstrating its potential for making reliable predictions.

The developed QL‐framework was first applied to derive a dose‐adaptive protocol for givinostat suitable for all the PV patients. To this end, a unique QL‐agent (QL_pop_‐agent) was trained on a heterogeneous population of 98 virtual patients. During the training phase, the designed reward function (Equations (1), (2), (3), (4), (5), (6), (7), (8), (9), (10), (11)) was refined by introducing smoothed penalties for severe toxicities (Equations (16), (17), (18) and Figures S1.1 and S1.2) to increase the efficacy of QL_pop_‐agent dosing protocol. QL_pop_‐agent was benchmarked against the givinostat clinical protocol on 10 test sets, each composed by a population of 98 virtual PV patients (Figure 3, Panel A). Both training and test populations were generated through a stratified random strategy Data (S3) in order to consider a broad spectrum of treatment response patterns and increase robustness of the QL‐agent with respect to IIV, a central feature in precision dosing. This strategy is particularly efficient also from a computational perspective as it allows to maximize the heterogeneity of the training set, maintaining a limited population size. The QL_pop_‐agent and givinostat clinical protocol showed similar efficacy, even if the QL_pop_‐agent had a bit more difficulties than the clinical protocol in avoiding severe toxicity events. Notably, the optimal dosing policy estimated by the QL_pop_‐agent confirmed the choice of the clinical protocol of maintaining the previous dose level when hematological parameters are in their efficacy ranges (i.e., $\mathrm{PLT}\in \left[150,400\right]\times 10^{9}/L$, $\mathrm{WBC}\in \left[4,10\right]\times 10^{9}/L$, HCT<45%). This nontrivial result is a confirmation of the powerfulness of RL algorithms that are able to learn from scratch a set of reasonable rules close to those formulated on the basis of the clinical knowledge.

However, population QL‐agent as well as the clinical protocol struggled to manage the presence of three endpoints to be simultaneously optimized and the high IIV, as both strategies are not tailored to each subject. These findings suggested that givinostat treatment can benefit from a deeper personalization of its dose‐adaptive protocol and that RL can be exploited for this aim. A set of personal QL‐agent (QL_ind_‐agents), one for each patient, was trained and compared with the clinical and QL_pop_‐agent protocols. The QL_ind_‐agents were able to optimize the efficacy/safety balance of givinostat treatment, to simultaneously manage the presence of multiple endpoints as well as high IIV, thus outperforming both population dosing strategies. In particular, using a tailored starting dose was central for QL_ind_‐agents to quickly maximize all the efficacy/safety endpoints of givinostat treatment with few dose adjustments (Figures S5.6–S5.8). Interestingly, as shown in Data S7, patients with similar response patterns were characterized by similar QL‐based individual dosing protocols.

The excellent performances demonstrated by the QL_ind_‐agents confirmed the potential benefits of adopting an individual‐oriented RL approach to delivery precision dosing in a clinical setting. However, an RL‐based personalization of the treatment could potentially also improve the drug development process by avoiding attrition due to wrong dosage [7]. As an example, QL_ind_‐agents were used in this paper to optimize the percentage of CHR achieved at 8 months of treatment, which is one of the primary endpoints of a planned Phase III study (see Data S6). To this aim, the reward function was modified to inform the RL algorithm of this specific goal. A bonus term that renumerates actions leading the patient to CHR at the eighth month (Equations S6.1 and S6.2) was introduced in the reward function. The QL_ind_‐agents were able to induce a CHR after 8 months of treatment in the entire patient population, thus maximizing the success rate of the clinical study. This simple example underlines the flexibility of the RL algorithm, which can be adapted to optimize different aspects of a pharmacological treatment if the goal is correctly formalized through a suitable reward function. Indeed, small changes in the reward function led to increase in the QL_pop_‐agent performances and the maximization of the givinostat Phase III clinical trial endpoint with individual QL‐based dosing protocols. States and actions were not fine‐tuned in this case study; however, it is worth remarking that in some scenarios, it could be necessary to adjust them to achieve specific goals.

Although the promising results in delivering precision dosing, the hybrid framework developed here by combining RL and PK‐PD modeling presents some limitations. First, it required long computational times, at least in the implementation proposed here In particular, the adoption of NONMEM as a simulation tool for patient pharmacological response (Panel B of Figure 1) coupled with Matlab is suboptimal. The simultaneous training of 98 QL_ind_‐agents and a QL_pop_‐agent took 26 and 52 days on a Linux machine with 8 i7 Intel cores (3.6 GHz of clock frequency), respectively. The use of different simulation tools can help to overcome this issue [32]. Second, analogously to what was proposed in [32], it was hypothesized that the PK‐PD model represents a digital twin of the patient and that it perfectly describes givinostat pharmacological response, as RUV was not considered in simulations. Neglecting RUV is a significant simplification that was necessary to obtain interpretable results in the presence of a complex scenario with three biomarkers to control. It is well established that the effectiveness of RL algorithms potentially decreases when RUV is introduced [19], making it essential to manage RUV appropriately based on its underlying source (i.e., model misspecification or measurement errors). In the first case, the reward function would no longer be deterministic, and it could be necessary to switch from classic RL algorithms (like QL) to methods specifically designed to handle the additional stochastic layer [18, 44]. Conversely, if RUV primarily represents measurement error, a shift from MDP to Partially Observable Markov Decision Process (POMDP) could provide a possible strategy to formally describe the uncertainty of the system state due to RUV [18, 44, 45]. Novel strategies to address these topics are currently still under investigation [44]. Third, it was hypothesized that the digital twin of each PV patient was known from the beginning of the treatment (i.e., all the individual parameters of the model are known without uncertainty). Actually, individual parameter estimation requires the availability of individual data, thereby necessitating real‐time monitoring during the treatment. A Bayesian approach combined with RL‐based treatment optimization can be used to progressively learn individual model parameters throughout the therapy monitoring, allowing for continuous refinement of the suggested dosing protocol [31, 46]. In this setup, at each monitoring step, the individual PK/PD model serving as the patient digital twin, along with the personal QL‐agent, should be updated, accounting for new patient data. This requires the personal QL‐agent to be retrained as patient data become available. As this process relies heavily on model simulations, it can be highly time‐consuming in the presence of a complex modeling framework. To ensure that the RL recommendations can be used in the clinical setting, the duration of the retraining process must fit within the time frame between patient observation and clinical decision. Consequently, an efficient implementation of the algorithm and modeling framework on a high‐performance computing architecture is essential [17, 32]. Finally, although the RL‐based precision dosing framework we developed for givinostat is grounded in a realistic clinical context and has shown promising results in simulation, it currently lacks validation on real clinical data. Therefore, once the previously described technical limitations are addressed, a rigorous clinical evaluation through randomized clinical trials will be essential to confirm that the benefits observed in simulations can be replicated in real‐world clinical settings. To the best of our knowledge, all the model‐informed RL frameworks applied to precision dosing have so far been validated exclusively through in silico simulations, with only one exception [38]. Defining a clinical trial setup that meets current regulatory standards to evaluate model‐informed RL approaches for precision dosing remains one of the most important and challenging open issues [17, 47].

In conclusion, coupling PK‐PD with RL algorithms can provide a useful approach to support precision dosing and improve treatment personalization, even to cope with nontrivial toy examples. However, before actual clinical applications, some further refinements are required.

## Author Contributions

A.D.C., E.M.T., and P.M. designed the research. A.D.C., E.M.T., and P.M. performed the research. A.D.C., E.M.T., and P.M. wrote the manuscript.

## Conflicts of Interest

The authors declare no conflicts of interest.

## Supporting information


Data S1.

Data S2.

Data S3.

Data S4.

Data S5.

Data S6.

Data S7.

## References

[1] R. W. Peck , “Precision Medicine Is Not Just Genomics: The Right Dose for Every Patient,” Annual Review of Pharmacology and Toxicology 58, no. 1 (2018): 105–122, 10.1146/annurev-pharmtox-010617-052446. 28961067

[2] R. J. Tyson , C. C. Park , J. R. Powell , et al., “Precision Dosing Priority Criteria: Drug, Disease, and Patient Population Variables,” Frontiers in Pharmacology 11 (2020): 420, 10.3389/fphar.2020.00420. 32390828 PMC7188913

[3] T. M. Polasek , S. Shakib , and A. Rostami‐Hodjegan , “Precision Dosing in Clinical Medicine: Present and Future,” Expert Review of Clinical Pharmacology 11, no. 8 (2018): 743–746, 10.1080/17512433.2018.1501271. 30010447

[4] T. Buclin , Y. Thoma , N. Widmer , et al., “The Steps to Therapeutic Drug Monitoring: A Structured Approach Illustrated With Imatinib,” Frontiers in Pharmacology 11 (2020): 177, 10.3389/fphar.2020.00177. 32194413 PMC7062864

[5] K. Maxfield , L. Milligan , L. Wang , et al., “Proceedings of a Workshop: Precision Dosing: Defining the Need and Approaches to Deliver Individualized Drug Dosing in the Real‐World Setting,” Clinical Pharmacology & Therapeutics 109, no. 1 (2021): 25–28, 10.1002/cpt.1933. 32663324 PMC8063505

[6] K. Maxfield and I. Zineh , “Precision Dosing: A Clinical and Public Health Imperative,” JAMA 325, no. 15 (2021): 1505–1506, 10.1001/jama.2021.1004. 33760028

[7] B. Ribba , “Reinforcement Learning as an Innovative Model‐Based Approach: Examples From Precision Dosing, Digital Health and Computational Psychiatry,” Frontiers in Pharmacology 13 (2023): 1094281, 10.3389/fphar.2022.1094281. 36873047 PMC9981647

[8] B. Chakraborty and S. A. Murphy , “Dynamic Treatment Regimes,” Annual Review of Statistics and Its Application 1 (2014): 447–464, 10.1146/annurev-statistics-022513-115553. PMC4231831 25401119

[9] J. S. Kang and M. H. Lee , “Overview of Therapeutic Drug Monitoring,” Korean Journal of Internal Medicine 24, no. 1 (2009): 1–10, 10.3904/kjim.2009.24.1.1. 19270474 PMC2687654

[10] D. Chan , V. Ivaturi , and J. Long‐Boyle , “The Time Is Now: Model‐Based Dosing to Optimize Drug Therapy,” International Journal of Pharmacokinetics 2, no. 4 (2017): 213–215, 10.4155/ipk-2017-0011.

[11] A. S. Darwich , T. M. Polasek , J. K. Aronson , et al., “Model‐Informed Precision Dosing: Background, Requirements, Validation, Implementation, and Forward Trajectory of Individualizing Drug Therapy,” Annual Review of Pharmacology and Toxicology 61, no. 1 (2021): 225–245, 10.1146/annurev-pharmtox-033020-113257. 33035445

[12] A. S. Darwich , K. Ogungbenro , A. A. Vinks , et al., “Why Has Model‐Informed Precision Dosing Not Yet Become Common Clinical Reality? Lessons From the Past and a Roadmap for the Future,” Clinical Pharmacology & Therapeutics 101, no. 5 (2017): 646–656, 10.1002/cpt.659. 28182269

[13] M. McComb , R. Bies , and M. Ramanathan , “Machine Learning in Pharmacometrics: Opportunities and Challenges,” British Journal of Clinical Pharmacology 88, no. 4 (2022): 1482–1499, 10.1111/bcp.14801. 33634893

[14] L. Hutchinson , B. Steiert , A. Soubret , et al., “Models and Machines: How Deep Learning Will Take Clinical Pharmacology to the Next Level,” CPT: Pharmacometrics & Systems Pharmacology 8, no. 3 (2019): 131–134, 10.1002/psp4.12377. 30549240 PMC6430152

[15] A. Chaturvedula , S. Calad‐Thomson , C. Liu , M. Sale , N. Gattu , and N. Goyal , “Artificial Intelligence and Pharmacometrics: Time to Embrace, Capitalize, and Advance?,” CPT: Pharmacometrics & Systems Pharmacology 8, no. 7 (2019): 440–443, 10.1002/psp4.12418. 31006175 PMC6657004

[16] B. Ribba , S. Dudal , T. Lavé , and R. W. Peck , “Model‐Informed Artificial Intelligence: Reinforcement Learning for Precision Dosing,” Clinical Pharmacology & Therapeutics 107, no. 4 (2020): 853–857, 10.1002/cpt.1777. 31955414

[17] E. M. Tosca , A. De Carlo , D. Ronchi , and P. Magni , “Model‐Informed Reinforcement Learning for Enabling Precision Dosing via Adaptive Dosing,” Clinical Pharmacology & Therapeutics 116, no. 3 (2024): 619–636, 10.1002/cpt.3356. 38989560

[18] R. S. Sutton and A. G. Barto , Reinforcement Learning: An Introduction (MIT press, 2018).

[19] B. Ribba , D. S. Bräm , P. G. Baverel , and R. W. Peck , “Model Enhanced Reinforcement Learning to Enable Precision Dosing: A Theoretical Case Study With Dosing of Propofol,” CPT: Pharmacometrics & Systems Pharmacology 11, no. 11 (2022): 1497–1510, 10.1002/psp4.12858. 36177959 PMC9662205

[20] M. Komorowski , L. A. Celi , O. Badawi , A. C. Gordon , and A. A. Faisal , “The Artificial Intelligence Clinician Learns Optimal Treatment Strategies for Sepsis in Intensive Care,” Nature Medicine 24, no. 11 (2018): 1716–1720, 10.1038/s41591-018-0213-5. 30349085

[21] C. Barbieri , M. Molina , P. Ponce , et al., “An International Observational Study Suggests That Artificial Intelligence for Clinical Decision Support Optimizes Anemia Management in Hemodialysis Patients,” Kidney International 90, no. 2 (2016): 422–429, 10.1016/j.kint.2016.03.036. 27262365

[22] A. Coronato , M. Naeem , G. De Pietro , and G. Paragliola , “Reinforcement Learning for Intelligent Healthcare Applications: A Survey,” Artificial Intelligence in Medicine 109 (2020): 101964, 10.1016/j.artmed.2020.101964. 34756216

[23] S. Liu , K. C. See , K. Y. Ngiam , L. A. Celi , X. Sun , and M. Feng , “Reinforcement Learning for Clinical Decision Support in Critical Care: Comprehensive Review,” Journal of Medical Internet Research 22, no. 7 (2020): e18477, 10.2196/18477. 32706670 PMC7400046

[24] W. J. Yun , M. Shin , S. Jung , J. Ko , H.‐C. Lee , and J. Kim , “Deep Reinforcement Learning‐Based Propofol Infusion Control for Anesthesia: A Feasibility Study With a 3000‐Subject Dataset,” Computers in Biology and Medicine 156 (2023): 106739, 10.1016/j.compbiomed.2023.106739. 36889025

[25] S. Anzabi Zadeh , W. N. Street , and B. W. Thomas , “Optimizing Warfarin Dosing Using Deep Reinforcement Learning,” Journal of Biomedical Informatics 137 (2023): 104267, 10.1016/j.jbi.2022.104267. 36494060

[26] A. E. Gaweda , E. D. Lederer , and M. E. Brier , “Artificial Intelligence–Guided Precision Treatment of Chronic Kidney Disease–Mineral Bone Disorder,” CPT: Pharmacometrics & Systems Pharmacology 11, no. 10 (2022): 1305–1315, 10.1002/psp4.12843. 35920131 PMC9574726

[27] R. Padmanabhan , N. Meskin , and W. M. Haddad , “Reinforcement Learning‐Based Control of Drug Dosing for Cancer Chemotherapy Treatment,” Mathematical Biosciences 293 (2017): 11–20, 10.1016/j.mbs.2017.08.004. 28822813

[28] G. Yauney , F. Doshi‐Velez , J. Fackler , et al., “Reinforcement Learning With Action‐Derived Rewards for Chemotherapy and Clinical Trial Dosing Regimen Selection,” in Proceedings of the 3rd Machine Learning for Healthcare Conference. Proceedings of Machine Learning Research. 85, 161–226, https://proceedings.mlr.press/v85/yauney18a.html (2018).

[29] P. Yazdjerdi , N. Meskin , M. Al‐Naemi , A.‐E. Al Moustafa , and L. Kovács , “Reinforcement Learning‐Based Control of Tumor Growth Under Anti‐Angiogenic Therapy,” Computer Methods and Programs in Biomedicine 173 (2019): 15–26, 10.1016/j.cmpb.2019.03.004. 31046990

[30] A. Ebrahimi Zade , S. Shahabi Haghighi , and M. Soltani , “Reinforcement Learning for Optimal Scheduling of Glioblastoma Treatment With Temozolomide,” Computer Methods and Programs in Biomedicine 193 (2020): 105443, 10.1016/j.cmpb.2020.105443. 32311510

[31] C. Maier , N. Hartung , C. Kloft , W. Huisinga , and J. de Wiljes , “Reinforcement Learning and Bayesian Data Assimilation for Model‐Informed Precision Dosing in Oncology,” CPT: Pharmacometrics & Systems Pharmacology 10, no. 3 (2021): 241–254, 10.1002/psp4.12588. 33470053 PMC7965840

[32] A. De Carlo , E. M. Tosca , M. Fantozzi , and P. Magni , “Reinforcement Learning and PK‐PD Models Integration to Personalize the Adaptive Dosing Protocol of Erdafitinib in Patients With Metastatic Urothelial Carcinoma,” Clinical Pharmacology & Therapeutics 115, no. 4 (2024): 825–838, 10.1002/cpt.3176. 38339803

[33] H. T. Chifotides , P. Bose , and S. Verstovsek , “Givinostat: An Emerging Treatment for Polycythemia Vera,” Expert Opinion on Investigational Drugs 29, no. 6 (2020): 525–536, 10.1080/13543784.2020.1761323. 32693648 PMC7534842

[34] E. M. Tosca , A. De Carlo , R. Bartolucci , et al., “In Silico Trial for the Assessment of Givinostat Dose Adjustment Rules Based on the Management of Key Hematological Parameters in Polycythemia Vera Patients,” CPT: Pharmacometrics & Systems Pharmacology 13, no. 3 (2024): 359–373, 10.1002/psp4.13087. 38327117 PMC10941510

[35] R. Marchioli , G. Finazzi , R. Landolfi , et al., “Vascular and Neoplastic Risk in a Large Cohort of Patients With Polycythemia Vera,” Journal of Clinical Oncology: Official Journal of the American Society of Clinical Oncology 23, no. 10 (2005): 2224–2232, 10.1200/JCO.2005.07.062. 15710945

[36] A. Iurlo , D. Cattaneo , C. Bucelli , and L. Baldini , “New Perspectives on Polycythemia Vera: From Diagnosis to Therapy,” International Journal of Molecular Sciences 21, no. 16 (2020): 5805, 10.3390/ijms21165805. 32823537 PMC7461104

[37] B. L. Stein , K. Patel , R. Scherber , J. Yu , D. Paranagama , and C. B. Miller , “MPN‐133: Mortality and Causes of Death of Patients With Polycythemia Vera: Analysis of the REVEAL Prospective, Observational Study,” Clinical Lymphoma Myeloma and Leukemia 21 (2021): S355, 10.1016/S2152-2650(21)01820-6.

[38] B. L. Moore , L. D. Pyeatt , V. Kulkarni , P. Panousis , K. Padrez , and A. G. Doufas , “Reinforcement Learning for Closed‐Loop Propofol Anesthesia: A Study in Human Volunteers,” Journal of Machine Learning Research 15, no. 1 (2014): 655–696.

[39] P. Magni and R. Bellazzi , “DT‐Planner: An Environment for Managing Dynamic Decision Problems,” Computer Methods and Programs in Biomedicine 54, no. 3 (1997): 183–200, 10.1016/s0169-2607(97)00044-8. 9421664

[40] A. Hochhaus , M. Baccarani , R. T. Silver , et al., “European LeukemiaNet 2020 Recommendations for Treating Chronic Myeloid Leukemia,” Leukemia 34, no. 4 (2020): 966–984, 10.1038/s41375-020-0776-2. 32127639 PMC7214240

[41] Matlab , “R2021a,” 2021 Natick, Massachussetts, MathWorks Inc.

[42] D. Ronchi , E. M. Tosca , R. Bartolucci , and P. Magni , “Go Beyond the Limits of Genetic Algorithm in Daily Covariate Selection Practice,” Journal of Pharmacokinetics and Pharmacodynamics 51, no. 2 (2024): 109–121, 10.1007/s10928-023-09875-7. 37493851 PMC10982092

[43] J.‐N. Eckardt , K. Wendt , M. Bornhäuser , and J. M. Middeke , “Reinforcement Learning for Precision Oncology,” Cancers 13, no. 18 (2021): 4624, 10.3390/cancers13184624. 34572853 PMC8472712

[44] A. De Carlo , E. M. Tosca , and P. Magni , Dealing With Stochasticity in Precision Dosing Decision‐Making Processes by Fully Exploiting PK‐PD Modelling in Reinforcement Learning Algorithms. A Practical Case‐Study on Vancomycin Continuous Infusion in ICU Patients, vol. 32 (Page, 2024).

[45] G. E. Monahan , “State of the Art—A Survey of Partially Observable Markov Decision Processes: Theory, Models, and Algorithms,” Management Science 28, no. 1 (1982): 1–16, 10.1287/mnsc.28.1.1.

[46] S. G. Wicha , A. G. Märtson , E. I. Nielsen , et al., “From Therapeutic Drug Monitoring to Model‐Informed Precision Dosing for Antibiotics,” Clinical Pharmacology & Therapeutics 109, no. 4 (2021): 928–941, 10.1002/cpt.2202. 33565627

[47] J. W. Ayers , N. Desai , and D. M. Smith , “Regulate Artificial Intelligence in Health Care by Prioritizing Patient Outcomes,” JAMA 331, no. 8 (2024): 639–640, 10.1001/jama.2024.0549. 38285467

