# Supplementary material for: Precision Dosing in Presence of Multiobjective Therapies by Integrating Reinforcement Learning and PK‐PD Models: Application to Givinostat Treatment of Polycythemia Vera
Source: CPT Pharmacometrics Syst Pharmacol. 2025 May 5;14(6):1018–31. doi: 10.1002/psp4.70012 (PMC12167923; doi:10.1002/psp4.70012)
Supplement: Supplementary file 1 — Data S1. [file PSP4-14-1018-s003.pdf]

Supplementary Materials S1

Reinforcement Learning

RL includes a set of algorithms to solve sequential decision-making problems, which can be formalized through a Markov Decision Process (MDP) [1].

In a MDP an agent interacts with a system to learn the optimal set of decision rules that drive the system towards a target condition. The agent has a finite set of possible actions, $A = \{a_1, \cdots, a_M\}$, and the system is described by a finite set of states, $S = \{s_1, \cdots, s_N\}$. Agent-system interplay (Figure 1, panel A) occurs at each time step of a given time sequence $t = 0,1,2, \cdots, T$. In particular, at each t, given the current observation of system state, $S_t = s$, the agent selects an action, $A_t = a$. This state-action association is formally represented by a function called policy, $\pi(s) = a$. Because of the agent action, at $t + 1$, the system evolves to a new state, S_{t+1}, and returns a reward signal, R_{t+1}, to the agent. The reward is a real number returned by a function, potentially depending on S_t, S_{t+1}, A_t, which mathematically describes the agent goal. Thus, R_{t+1} quantifies the appropriateness of the agent policy (i.e., performing action a when the system is in the state s) with respect to the final goal.

As each agent action produces a reward, the entire decisional process is characterized by the reward sequence, $R_{t+1}, R_{t+2}, \cdots R_T$, which are higher if the performed actions are useful to reach the target. Therefore, in the MDP formalism, achieving system target condition means maximizing the discounted return, G_t (Eq.1), which depends on the rewards collected in all the subsequent time steps.

$$G_t = R_{t+1} + \gamma R_{t+2} + \gamma^2 R_{t+3} + \cdots = \sum_{k=0}^{T} \gamma^k R_{t+k+1}$$

(S1. 1)

The parameter $\gamma \in (0,1)$, named discount factor, ensures the convergence of G_t for $T \to \infty$ and regulates the importance given to future rewards with respect the present one. If γ is close to 0, agent will maximize only immediate reward, conversely, if $\gamma \to 1$, agent becomes more farsighted.

Solving a MDP means finding the optimal policy, $\pi^*(s) = a^*$, that maximizes G_t by selecting the most appropriate action, a^*, for each system state, s. To this end, RL algorithms leverage a utility function, called Q-function or action-value function, $Q^\pi(s, a)$. By definition (Eq.2), $Q^\pi(s, a)$ is the conditional expected value of G_t given the choice of action a when system state is s, according to the policy π.

$$Q^\pi(s, a) := \mathbb{E}_\pi[G_t | S_t = s, A_t = a] = \mathbb{E}_\pi\left[\sum_{k=0}^{T} \gamma^k R_{t+k+1} | S_t = s, A_t = a\right].$$

(S1. 2)

Once the Q-function is estimated for each state-action couple, π^* can be derived through Eq.S1.3.

$$\pi^*(s) = a^* = \arg\max_a Q^\pi(s, a).$$

(S1. 3)

In this work, Q-Learning (QL) algorithm [1] was used to solve the MDP associated with the givinostat adaptive dosing treatment. A comprehensive description of QL and its training strategy is reported in the section below.

QL Algorithm

QL is a RL algorithm that estimates the optimal policy directly from experience, i.e., by collecting sequences of transitions $< S_t, A_t, S_{t+1}, R_{t+1} >$. In particular, QL relies on a tabular approximation of the Q-function for each state-action pair. Given a set of N states and M actions, QL returns a matrix, $\mathbf{Q} \in \mathbb{R}^{N\times M}$, in which the element $Q[s_i, a_j]$ represents an approximation of the Q-function for the action a_j in state s_i. This means that the QL algorithm requires the state-action space to be discrete and small to produce accurate estimations. The main advantage of QL algorithm is an easy translation of the **Q**-matrix into a set of human-readable if-then-else statements as illustrated in Figure S1.1.

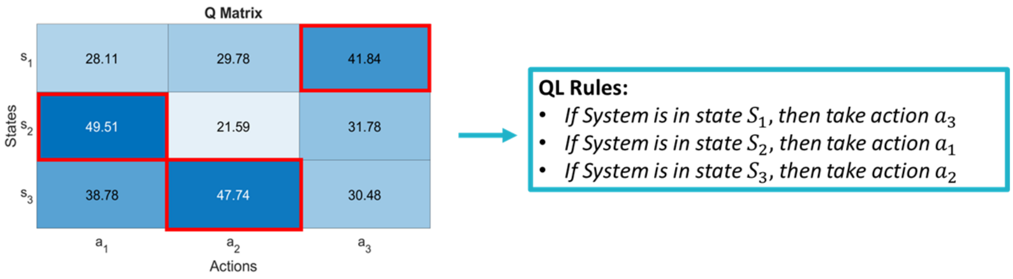

Figure S1. 1*:* Translating into human readable "if-then-else" rules Q matrix optimal policy.

As reported in Listing 1, the learning of the **Q**-matrix is an iterative process where a single episode composed by T steps is repeated for I times. Starting from an arbitrary matrix, at each time step t, the entry $Q[S_t, A_t]$ of the matrix is updated with:

$$\mathbf{Q}[S_t, A_t] \leftarrow \mathbf{Q}[S_t, A_t] + \alpha \left[R_{t+1} + \gamma \arg \max_{a} \mathbf{Q}[S_{t+1}, A_t] - \mathbf{Q}[S_t, A_t] \right]$$

(S1.4)

where α is the learning rate that weighs the magnitude of the updates.
QL training is applied with a ε-greedy strategy to address the exploitation/exploration dilemma, i.e., the agent should not exploit only the currently estimated best policy but should also keep exploring the state-action space in order to potentially discover better strategies. When the ε-greedy strategy is adopted in QL training, at each time step t, the agent has a given probability ε of taking a random action instead of the best estimated action by using Eq. S1.3.

Algorithm 1: Pseudocode for Q-Learning algorithm.

Given: set of N states, set of M actions, learning rate α, discount factor γ, a probability ϵ, a maximum number of training iterations I, probability ϵ
init Q matrix arbitrarily
loop for each episode (I times):
Set the current system state to S_0
loop for each decisional time step t:
p ← uniform random number $\in [0,1]$
if p<ϵ
    Select action A_t randomly
  else
    $A_t \leftarrow \arg\max_a Q(S_t, a)$
Perform A_t on the system

Observe next state S_{t+1} and reward R_{t+1}

$$Q(S_t, A_t) \leftarrow Q(S_t, A_t) + \alpha \cdot [R_{t+1} + \gamma \cdot \max_a Q(S_{t+1}, a) - Q(S_t, A_t)]$$

Set current system state to S_{t+1}

Reward function for QL_{pop}-agent

Figures S1.2-S1.3 illustrates the reward function defined for the QL_{pop}-agent with a smoothed penalty (Eq.19-21) in case of severe toxicities (i.e., $PLT_{Obs} < 75 \times 10^9/L$ and/or $WBC_{Obs} < 3 \times 10^9/L$). Differently from the original formulation (Eq.4-14) which assigned a reward equal to 0 (i.e., the minimum value assumed by the proposed reward function), in this case an exponential decay of the reward was adopted as the observed values of PLT and WBC go below $75 \times 10^9/L$ and $3 \times 10^9/L$, respectively.

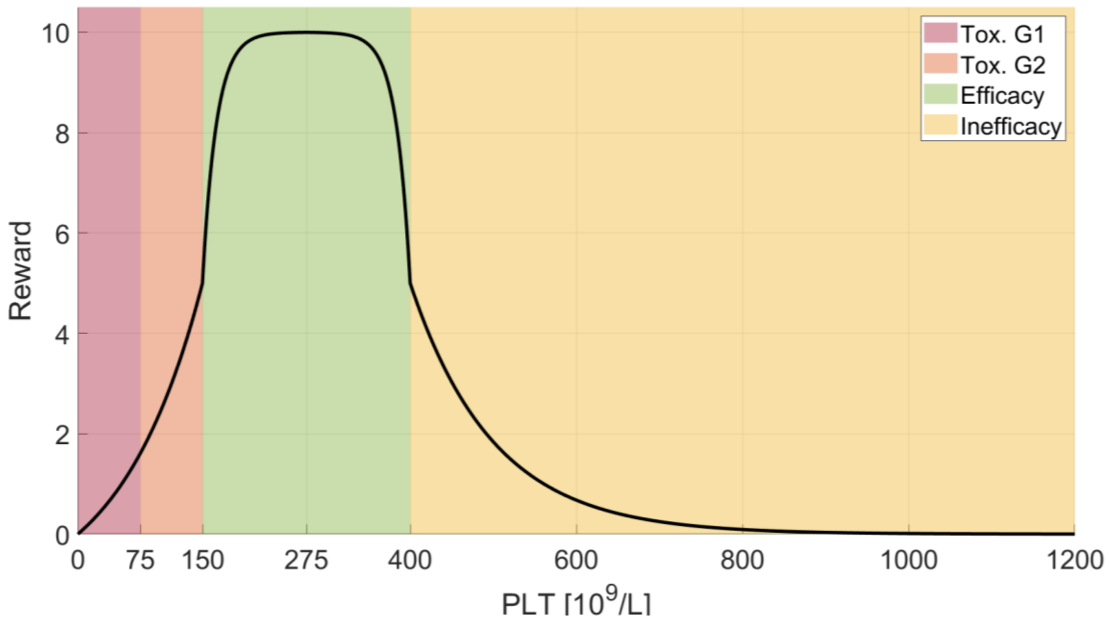

Figure S1. 2 Plot of the smoothed version of the $Reward_{PLT,Obs}$ function which evaluates the monitored values of PLT.

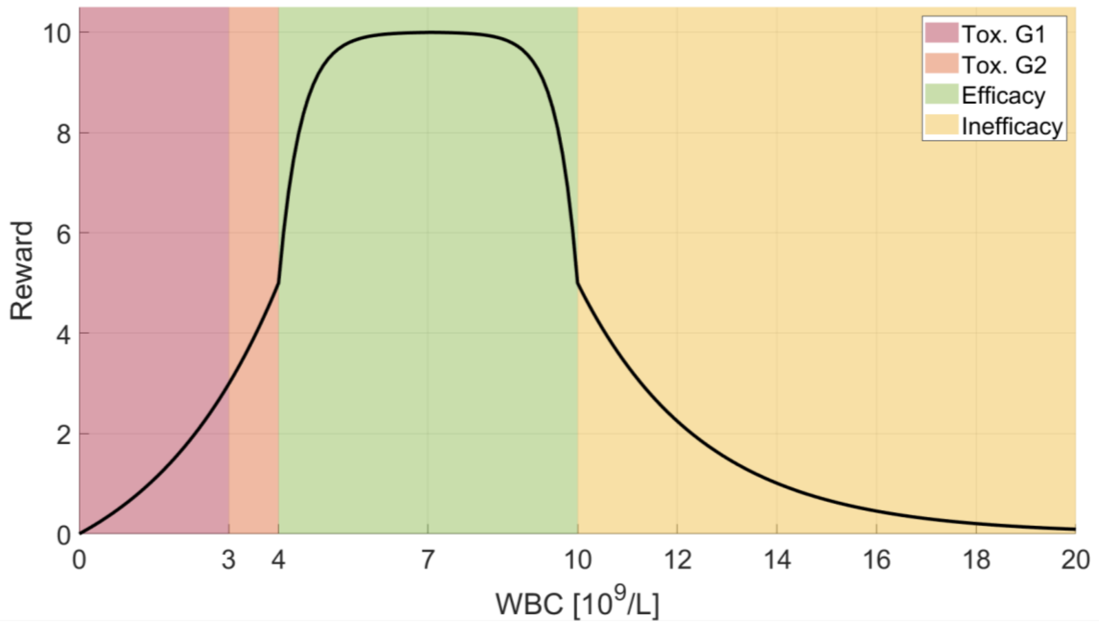

Figure S1. 3 Plot of the smoothed version of the $Reward_{WBC,Obs}$ function which evaluates the monitored values of WBC.

Terms in the Reward Function based on the derivative of the haematological parameters

Figures S1.4-S1.6 provide a representation of the terms in the reward function (Eq.2) based on the derivative, y', of the haematological parameters (i.e., $Reward_{PLT,Der}$, $Reward_{WBC,Der}$, $Reward_{HCT,Der}$, Eqs.12-14). In particular, representation of $Reward_{,Der}$ is stratified according to the efficacy (Fig. S1.4), toxicity (Fig. S1.5) and inefficacy (Fig.S1.6) range in which the haematological parameter falls. As in this case study a toxicity range was not defined for HCT, only functions in Figure S1.4 and Figure S1.6 were used for this haematological parameter.

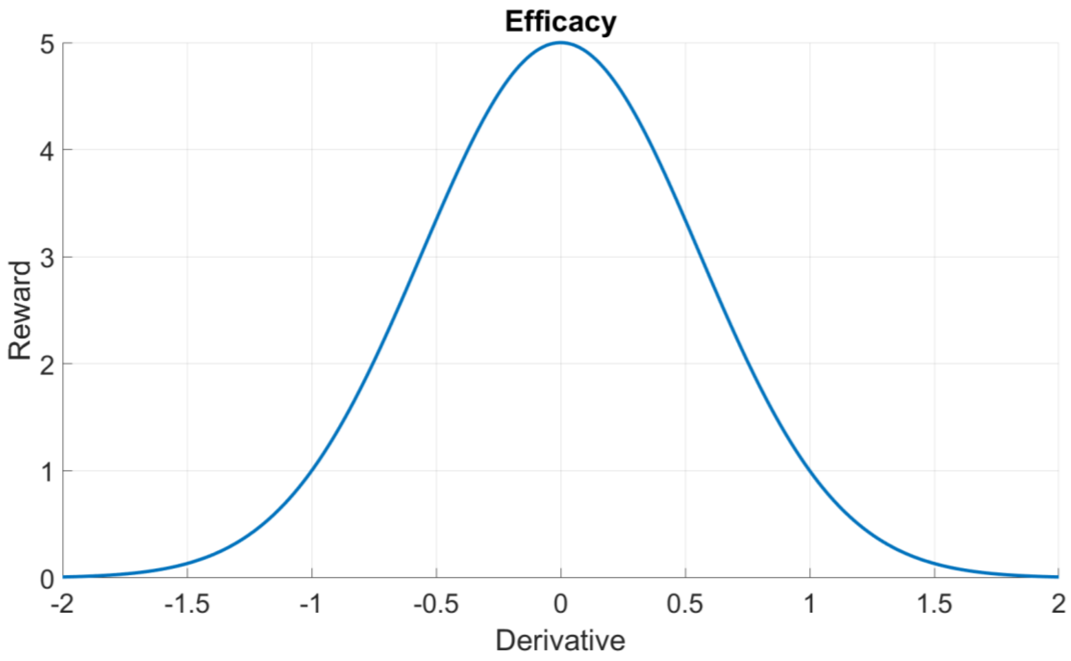

Figure S1. 4: Reward function adopted to evaluate the derivative of PLT, WBC and HCT when their values fall within the efficacy range (i.e., $PLT \in [150{,}400] \times 10^9/L, WBC \in [4{,}10] \times 10^9/L, HCT < 45\%$).

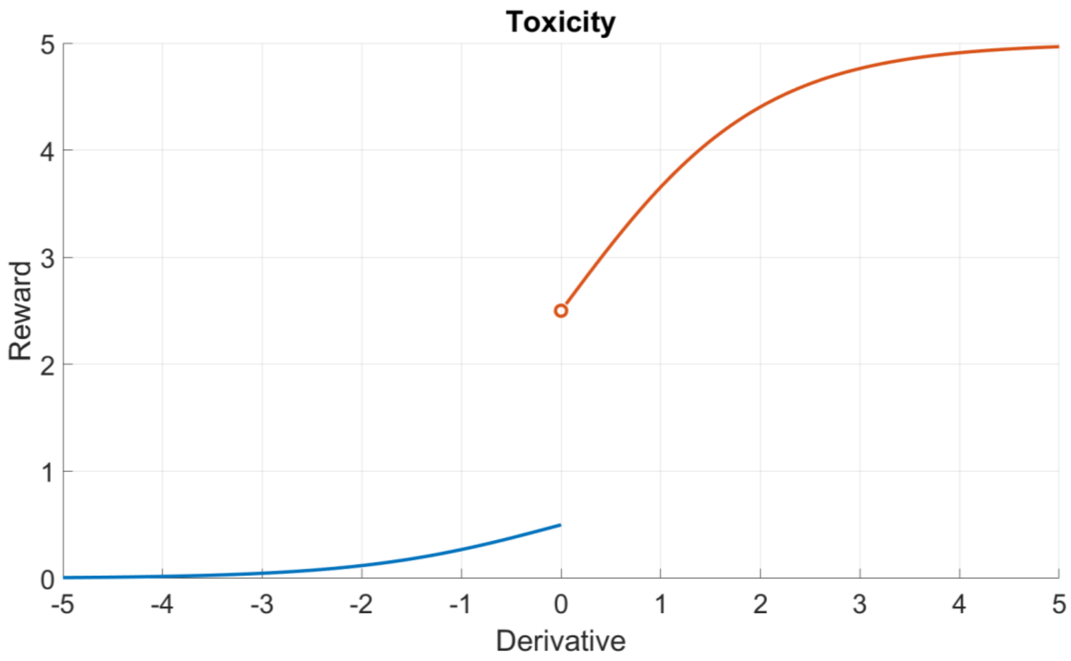

Figure S1. 5: Reward function adopted to evaluate the derivative of PLT and WBC when their values fall within the toxicity range (i.e., $PLT < 150 \times 10^9/L, WBC < 4 \times 10^9/L$). This function was not applied to HCT derivative as a toxicity range was not defined for this parameter.

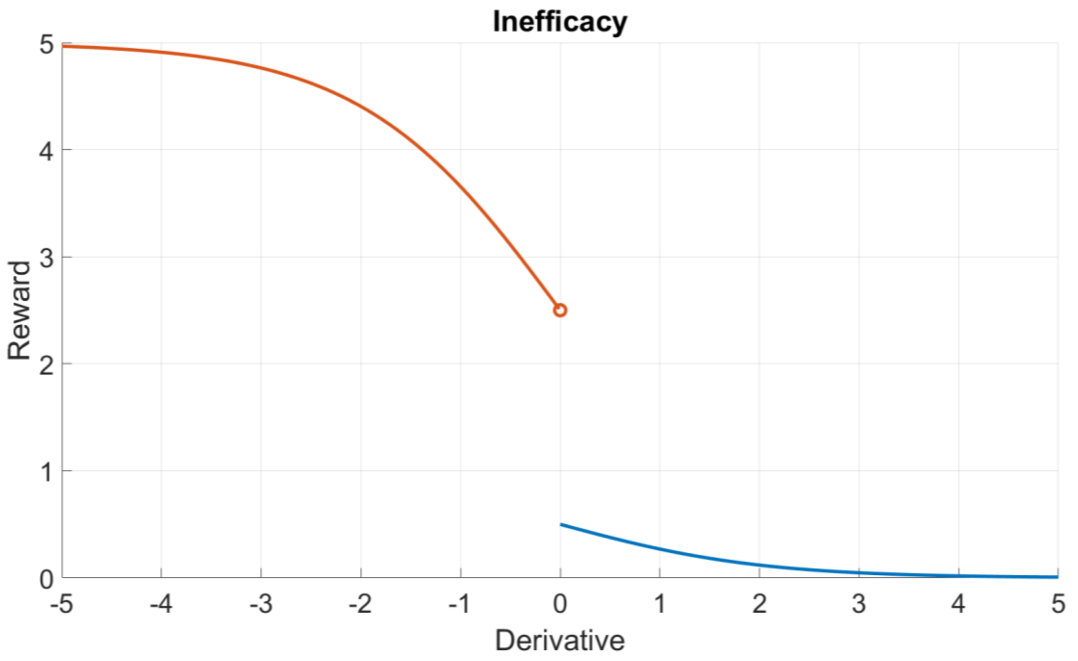

Figure S1. 6: Reward function adopted to evaluate the derivative of PLT, WBC and HCT when their values fall within the inefficacy range (i.e., $PLT > 400 \times 10^9/L, WBC\ > 10 \times 10^9/L, HCT \geq 45\%$).

Reward component evaluating the end-of-cycle observations of PLT, WBC and HCT

In this section, the plots representing the components of the reward function (Eq. 2 of the main text) evaluating the end-of-cycle observations i.e., $Reward_{PLT,Obs}, Reward_{WBC,Obs}$ and $Reward_{HCT,Obs}$ are reported.

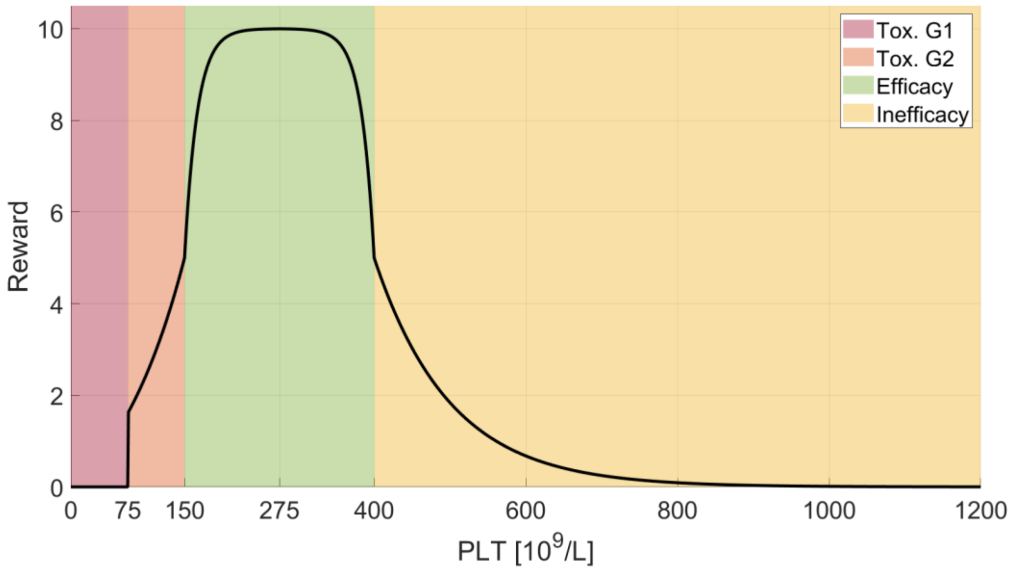

Figure S1.7: Plot of the $Reward_{PLT,Obs}$ function which evaluates the monitored values of PLT. Values are reported in the [0,10] scale according to its weight ($\beta_1\ = 10$) as reported in Eq. 2 of the main text.

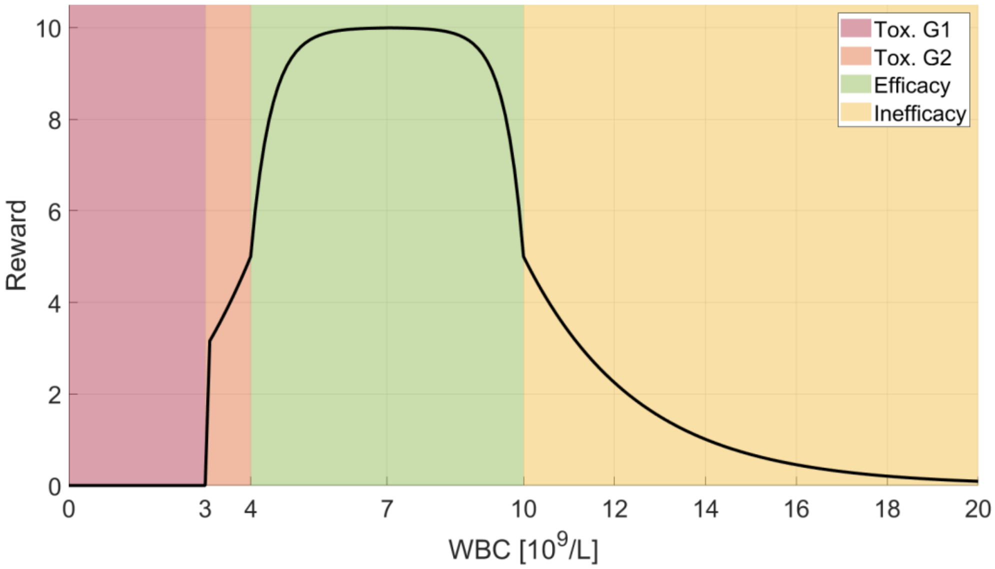

Figure S1.8: Plot of the $Reward_{WBC,Obs}$ function which evaluates the monitored values of PLT. Values are reported in the [0,10] scale according to its weight ($\beta_1 = 10$) as reported in Eq. 2 of the main text.

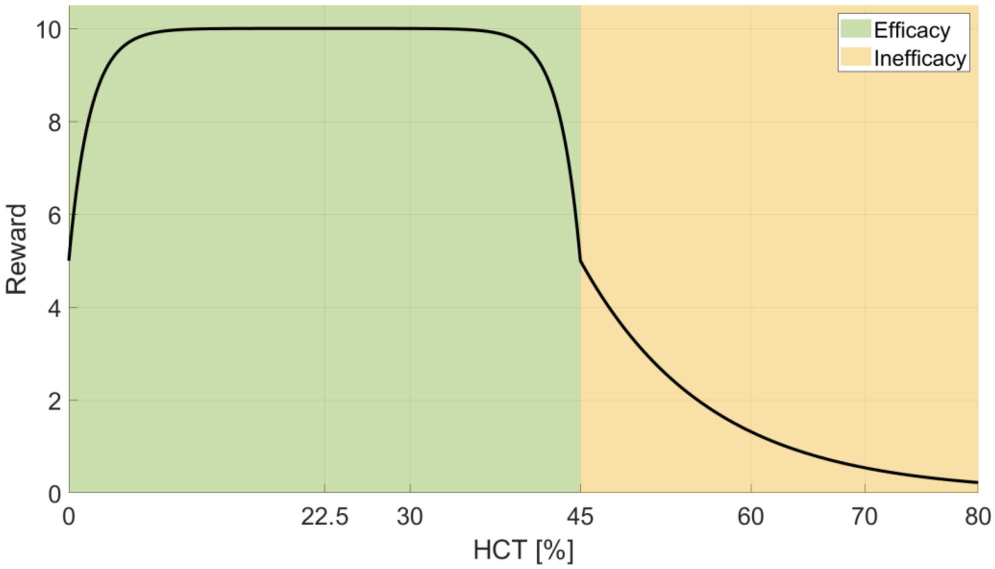

Figure S1. 9: Plot of the $Reward_{HCT,Obs}$ function which evaluates the monitored values of PLT. Values are reported in the [0,10] scale according to its weight ($\beta_1 = 10$) as reported in Eq. 2 of the main text.

References

[1] R. S. Sutton e A. G. Barto, *Reinforcement learning: An introduction*. MIT press, 2018.
