# Supplementary material for: Precision Dosing in Presence of Multiobjective Therapies by Integrating Reinforcement Learning and PK‐PD Models: Application to Givinostat Treatment of Polycythemia Vera
Source: CPT Pharmacometrics Syst Pharmacol. 2025 May 5;14(6):1018–31. doi: 10.1002/psp4.70012 (PMC12167923; doi:10.1002/psp4.70012)
Supplement: Supplementary file 2 — Data S2. [file PSP4-14-1018-s002.pdf]

Supplementary Materials S2

Population PK Model of givinostat

The PK model for givinostat is a two compartmental model with a lagged first order absorption for oral administration and elimination from central compartment [1]. Table S2.1 reports model parameters. A Log-Normal distribution was assumed for all the model parameters. Givinostat clearance (CL) is influenced by individual body weight scaled by median weight in the population which was equal to 77 Kg (Eq. S2.1).

$$CL_i = \left(\frac{Weight_i}{77}\right)^{\theta_{Weight}}$$

(S2. 1)

Table S2. 1 Parameter values of Givinostat population PK model.

**Parameter**	**Description**	**Unit**	**Value**	**Variance of IIV, Ω**
CL/F	Apparent clearance	L/h	181	0.083
V_2/F	Apparent volume of distribution of central compartment	L	171	0.490
Q/F	Apparent inter-compartment clearance	L/h	33.3	-
V_3/F	Apparent volume of distribution of peripheral compartment	L	491	0.070
K_A	Absorption rate	1/L	0.233	0.058
ALAG	Lag time	h	0.221	-
θ_{Weight}	Body Weight covariate effect	-	0.402	-

PK-PD Model for givinostat effect on PLT, WBC and HCT

Givinostat myelosuppressive effect on blood cells was described by a joint Friberg model for PLT, WBC and HCT as illustrated in Figure S2.1 and Eq. S2.1 [1,2]. The values of model parameters are reported in Table S2.2.

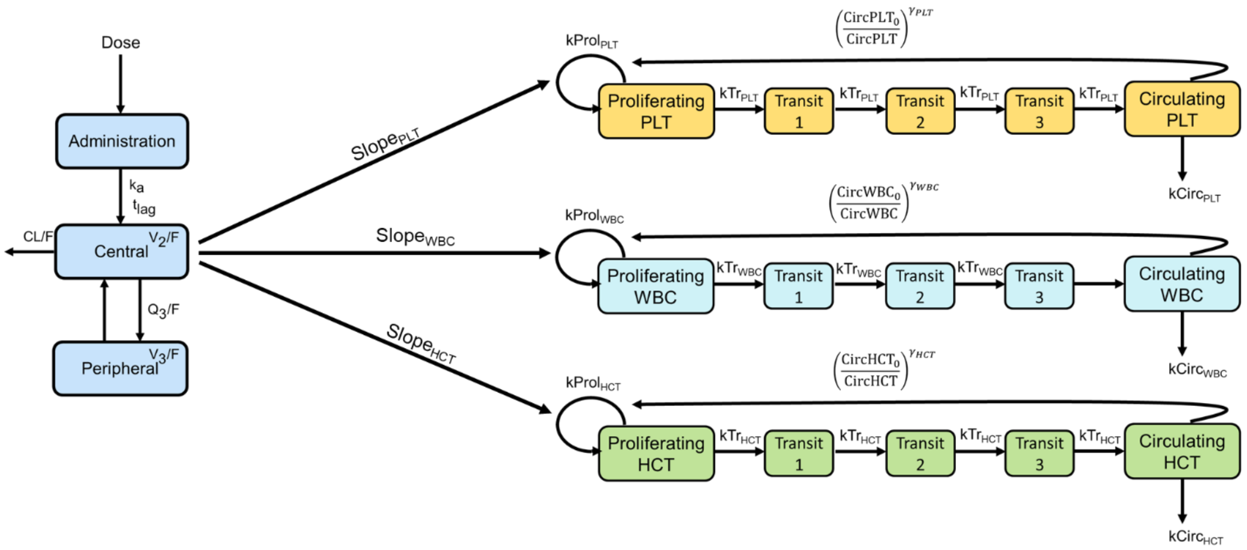

Figure S2. 1 Schematical representation of Givinostat population PK-PD model.

$$\frac{dProl(t)}{dt} = k_{prol} \cdot Prol(t) \cdot (1 - E_{drug}(t)) \cdot \left(\frac{Circ_0}{Circ(t)}\right)^{\gamma} - k_{tr} \cdot Prol(t)$$

$$\frac{dTransit_1(t)}{dt} = k_{tr} \cdot Prol(t) - k_{tr} \cdot Transit_1(t)$$

$$\frac{dTransit_2(t)}{dt} = k_{tr} \cdot Transit_1(t) - k_{tr} \cdot Transit_2(t)$$

$$\frac{dTransit_3(t)}{dt} = k_{tr} \cdot Transit_2(t) - k_{tr} \cdot Transit_3(t)$$

$$\frac{dCirc(t)}{dt} = k_{tr} \cdot Transit_3(t) - k_{circ} \cdot Circ(t)$$

$$E_{drug}(t) = Slope \cdot conc_p(t)$$

$$Prol(0) = Transit_1(0) = Transit_2(0) = Transit_3(0) = Circ_0$$

$$k_{tr} = k_{prol} = k_{circ} = \frac{N+1}{MTT} \text{ with } N = \text{number of transit compartment} = 3$$

(S2. 2)

Table S2. 2 Parameter values of Givinostat population PK-PD model

**Parameter**	**Description**	**PLT**	**WBC**	**HCT**
**Value**				
MTT	Mean Transit Time (h)	297	319	610
$Circ_0$	Steady state circulating level (10^9/L for PLT and WBC, % for HCT)	670	15	46.1
γ	Feedback	0.142	0.097	0.21
Slope	Drug potency (L/ng)	3.74	1.94	0.313
**Inter-Individual Variability (IIV), Ω**				
Omega MTT	Variance (CV%)	0.085 (29.7)	0.017 (13.0)	0.095 (31.5)

Omega $Circ_0$	Variance (CV%)	0.082 (29.2)		0.118 (35.3)		0.002 (4.78)	
Omega γ	Variance (CV%)	0.167 (42.6)		0.079 (28.6)		0 fixed	
Omega Slope	Variance (CV%)	0.199 (47.0)		0.142 (39.0)		1.27 (160)	
**Correlation Between IIV**							
Omega (2,1)	Covariance (Correlation) MTT_{PLT}/MTT_{WBC}	0.038 (1.000)		---	---	---	---
Omega (8,7)	Covariance (Correlation) $\gamma_{PLT}/\gamma_{WBC}$	0.1 (0.871)	49.0	---	---	---	---
Omega (11,10)	Covariance (Correlation) $Slope_{PLT}/Slope_{WBC}$	0.106 (0.628)	43.0	---	---	---	---
Omega (12,10)	Covariance (Correlation) $Slope_{PLT}/Slope_{HCT}$	0.238 (0.473)	49.7	---	---	---	---
Omega (12,11)	Covariance (Correlation) $Slope_{WBC}/Slope_{HCT}$	0.225 (0.530)	43.0	---	---	---	---

Steady State Analysis of Givinostat PK-PD Model

A steady-state analysis of the joint PK-PD Friberg model was performed to characterize *a priori* the response to Givinostat treatment for each patient. Assuming a constant concentration for Givinostat, i.e., $conc_p(t) = \bar{c}$, the equilibrium points of the system $\left(\overline{Prol}, \overline{Transit_1}, \overline{Transit_2}, \overline{Transit_3}, \overline{Circ}\right)$ can be derived zeroing the differential equations in Eq.S2.2. It follows that they have to satisfies the relationships:

$$\overline{Prol} = \overline{Transit_1} = \overline{Transit_2} = \overline{Transit_3} = \overline{Circ}$$

(S2. 3)

$$\left(\frac{Circ_0}{\overline{Circ}}\right)^{\gamma} = 1 - \bar{c} \cdot Slope.$$

(S2. 4)

These relationships hold for PLT, WBC and HCT. Considering the daily exposure of Givinostat, i.e., $AUC_{0-24h} = \bar{c} \cdot 24h = \overline{Dose}/CL$, we can express $\bar{c}$ as $\bar{c} = \overline{Dose}/(CL \cdot 24h)$, and, replacing it in Eq. S2.4, we obtain:

$$\overline{Dose} = CL \cdot 24h \cdot \frac{\left[1 - \left(\frac{Circ_0}{\overline{Circ}}\right)^{\gamma}\right]}{Slope}.$$

(S2. 5)

Therefore, given individual PK-PD parameters and the target range of each biomarker, Eq. S2.5 can be applied to compute the theorical dosing window leading to the complete haematological response. As

will be discussed in *Supplementary Materials S3*, this result will be adopted to define the virtual population on which the methodology will be applied.

References

[1] E. M. Tosca *et al.*, «In silico trial for the assessment of givinostat dose adjustment rules based on the management of key hematological parameters in polycythemia vera patients», *CPT: Pharmacometrics & Systems Pharmacology*, vol. 13, no. 3, feb. 2024, doi: 10.1002/psp4.13087.

[2] L.E. Friberg et al., «Model of chemotherapy-induced myelosuppression with parameter consistency across drugs», *Journal of Clinical Oncology*, vol. 2o, no. 24, pp.4713-4721, doi:10.1200/JCO.2002.02.140
