# Supplementary material for: Precision Dosing in Presence of Multiobjective Therapies by Integrating Reinforcement Learning and PK‐PD Models: Application to Givinostat Treatment of Polycythemia Vera
Source: CPT Pharmacometrics Syst Pharmacol. 2025 May 5;14(6):1018–31. doi: 10.1002/psp4.70012 (PMC12167923; doi:10.1002/psp4.70012)
Supplement: Supplementary file 3 — Data S3. [file PSP4-14-1018-s006.pdf]

# Supplementary Materials S3

## Distribution of Patients Covariates

As described in Supplementary Materials S2, the only covariate included in the Givinostat PK-PD model is body weight that affect Givinostat CL. To generate a plausible virtual population of PV patients, it was assumed that $WT \sim (77,16^2)$ according to distribution of body weight in PV patients on which the model was originally built [1].

## Generation of Virtual Populations of PV patients

The aim of this section is to provide a detailed description of the steps followed to generate the virtual populations of PV patients used as training and test sets for the QL-agents. The same procedure was followed for all the virtual populations. As illustrated in Panel B of Figure 3 and reported in the manuscript, the $QL_{pop}$-agent was trained on a pool of 98 virtual patients and then evaluated on 10 test sets of different PV patients sharing the same givinostat response dynamics. Differently the panel of $QL_{ind}$-Agents were trained and evaluated on the pool of patients used to train $QL_{pop}$-agent.

1. Given the distributions of PK-PD model parameters (*Supplementary Materials S2*) and patients covariates (section *Distribution of Patients Covariates*) an initial pool of 500000 virtual patients (i.e., 500000 sets of individual PK-PD model parameters and body weight) was randomly extracted.
2. For each virtual patient a theoretical dose window was defined for PLT, WBC and HCT with eq. (S2.5). In particular, lower and upper bounds of therapeutic ranges were obtained by replacing $\overline{Circ}$ with the efficacy range limits (i.e., $PLT \in [150{,}400] \times 10^9/L$, $WBC \in [4{,}10] \times 10^9/L$, $HCT < 45\%$).
3. The three dose ranges obtained for PLT, WBC and HCT, were used to classify PV patients in:
    a. **Theoretically responders** (57.10%): if the intersection of the dose windows of PLT, WBC and HCT is not-empty and includes levels lower than the maximum tolerated dose of 200 mg/day.
    b. **Theoretically not responders** (42.90%): if the intersection of the PLT, WBC and HCT dose windows:
        i. is empty;
        ii. is not empty but it includes only dose higher than 200 mg/day;
4. Under the assumption that an ineffective drug would never be selected for the treatment of a certain disease, only theoretically responders were considered for the analysis.
5. Theoretically responders were subdivided into 14 groups (Table S3.1) based on which available givinostat doses are included in the intersection range previously defined.

**Table S3. 1** Groups defined according to givinostat theoretical optimal dose.

| Group | Description |
|---|---|
| <50 | The upper bound of the intersection range is below 50 mg/day. |
| 50 | 50 mg/day is the only dose falling within the intersection range. |
| 75 | 75 mg/day is the only dose falling within the intersection range. |
| 100 | 100 mg/day is the only dose falling within the intersection range. |

| | |
|---|---|
| 125 | 125 mg/day is the only dose falling within the intersection range. |
| 150 | 150 mg/day is the only dose falling within the intersection range. |
| 175 | 175 mg/day is the only dose falling within the intersection range. |
| 200 | 200 mg/day is the only dose falling within the intersection range. |
| 50,75 | 50 and 75 mg/day are the only doses falling within the intersection range. |
| 75,100 | 75 and 100 mg/day are the only doses falling within the intersection range. |
| 100,125 | 100 and 125 mg/day are the only doses falling within the intersection range. |
| 125,150 | 125 and 150 mg/day are the only doses falling within the intersection range. |
| 150,175 | 150 and 175 mg/day are the only doses falling within the intersection range. |
| 175,200 | 175 and 200 mg/day are the only doses falling within the intersection range. |

6. A further stratification of theoretically responders was introduced according to their baseline characteristics (i.e., $Circ0_{PLT}, Circ0_{WBC}, Circ0_{HCT}$ parameters), as reported in Table S3.2. The condition with normal values of all $Circ0_{PLT}, Circ0_{WBC}$ and $Circ0_{HCT}$ is missing as all PV patients have at least one haematological parameter not in the target range at baseline [1].

**Table S3. 2** Groups defined according to PV patients baseline conditions.

| **Baseline characteristic within normality range (Yes/No)** | | | **Group** |
|---|---|---|---|
| $Circ0_{PLT}$ | $Circ0_{WBC}$ | $Circ0_{HCT}$ | |
| No | No | No | C1 |
| Yes | No | No | C2 |
| Yes | Yes | No | C3 |
| No | Yes | No | C4 |
| No | Yes | Yes | C5 |
| No | No | Yes | C6 |
| Yes | No | Yes | C7 |

7. The 7 groups defined in Table S3.2 were combined with the 14 categories listed in Table S3.1, thus defining 98 subgroups of theoretical respondent virtual patients.
8. As illustrated in Figure S3.1, each virtual population was obtained by randomly sampling one individual for each of the 98 subgroups.

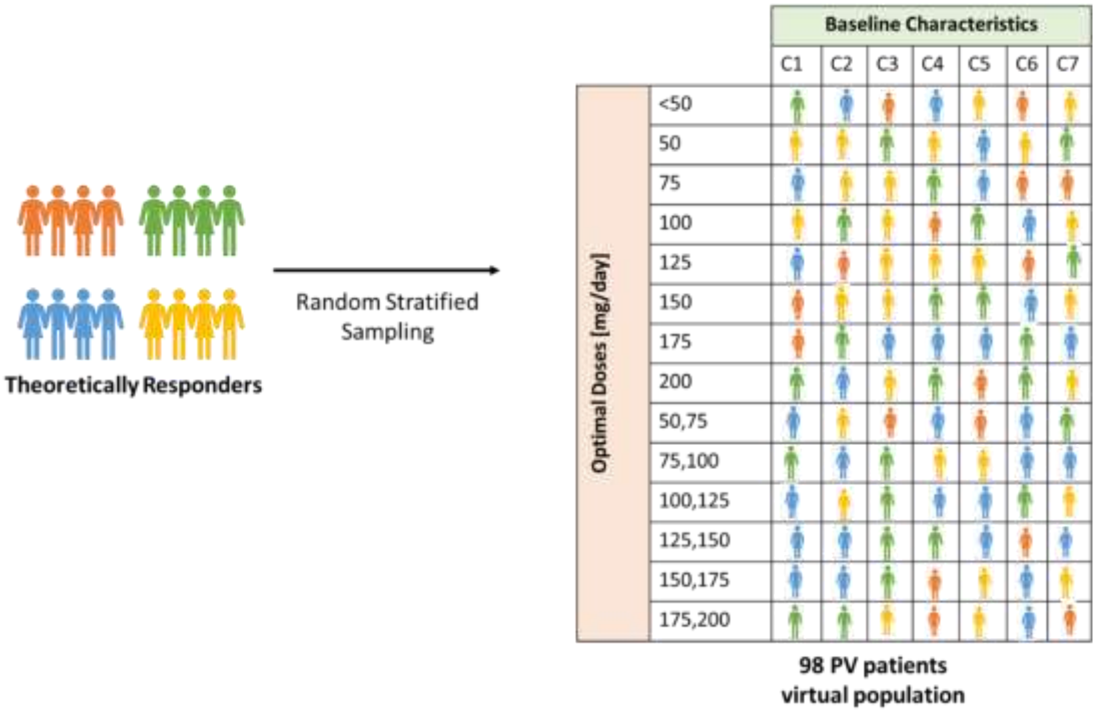


**Figure S3. 1** Stratified random sampling strategy for building the final virtual population.

## References

[1] E. M. Tosca *et al.*, «In silico trial for the assessment of givinostat dose adjustment rules based on the management of key hematological parameters in polycythemia vera patients», *CPT: Pharmacometrics & Systems Pharmacology*, vol. 13, no 3, feb. 2024, doi: 10.1002/psp4.13087.
