# Supplementary material for: Precision Dosing in Presence of Multiobjective Therapies by Integrating Reinforcement Learning and PK‐PD Models: Application to Givinostat Treatment of Polycythemia Vera
Source: CPT Pharmacometrics Syst Pharmacol. 2025 May 5;14(6):1018–31. doi: 10.1002/psp4.70012 (PMC12167923; doi:10.1002/psp4.70012)
Supplement: Supplementary file 4 — Data S4. [file PSP4-14-1018-s007.pdf]

Supplementary Materials S4

Hyperparamters of the QL Algorithm

Table S4. 1 Hyperparameters used to train the QL_{pop}-agent.

Hyperparameter	Value
Number of iterations	50000
Learning Rate	0.1
Discount Factor (γ)	0.99
Probability of ϵ-greedy strategy	$\epsilon = \max\left(0.3, \exp\left(i \cdot \left(-\frac{\ln 0.3}{10000}\right)\right)\right)$ with i being the current iteration number

Table S4. 2 Hyperparameters used to train the QL_{ind}-agents.

Hyperparameter	Value
Number of iterations	50000
Learning Rate	0.1
Discount Factor (γ)	0.97
Probability of ϵ-greedy strategy	$\epsilon = \max\left(0.3, \exp\left(i \cdot \left(-\frac{\ln 0.3}{10000}\right)\right)\right)$ with i being the current iteration number

Evaluation of the new Reward function for the QL_{pop}-agent

The aim of this section is to provide a comparison of the performances of the QL_{pop}-agent characterized by the first reward (Eqs.4-14, abbreviated here with Rew1) and by the second one with a smoother penalization on severe toxicities (Eqs.19-21, abbreviated here with Rew2). This assessment was performed on the training virtual population, which is the same in both the cases.

The effect of the two reward functions on the QL_{pop}-agent policies can be seen in Figure S4.1. For Rew1 (Panel A) and Rew2 (Panel B), the median (black line) and 90% C.I. (shaded blue area) of the individual profiles in the population are reported. Due to the strong penalties assigned to the severe toxicities in Rew1 (i.e., reward=0, Eq.4), the corresponding QL_{pop}-agent avoided the choice of higher dose levels and totally discouraged the use of the maximum tolerated dose (i.e., 200 mg/day) (see the distribution of each dose levels within the eight month timeframe reported in Figure S4.2). Form one hand, this conservative dose strategy prevented a longer permanence of PLT and WBC below $75 \times 10^9/L$ and $3 \times 10^9/L$ (red shaded area in Figure S4.1), respectively, from the other hand it was unable to satisfying bring the hematological parameters in the efficacy range.

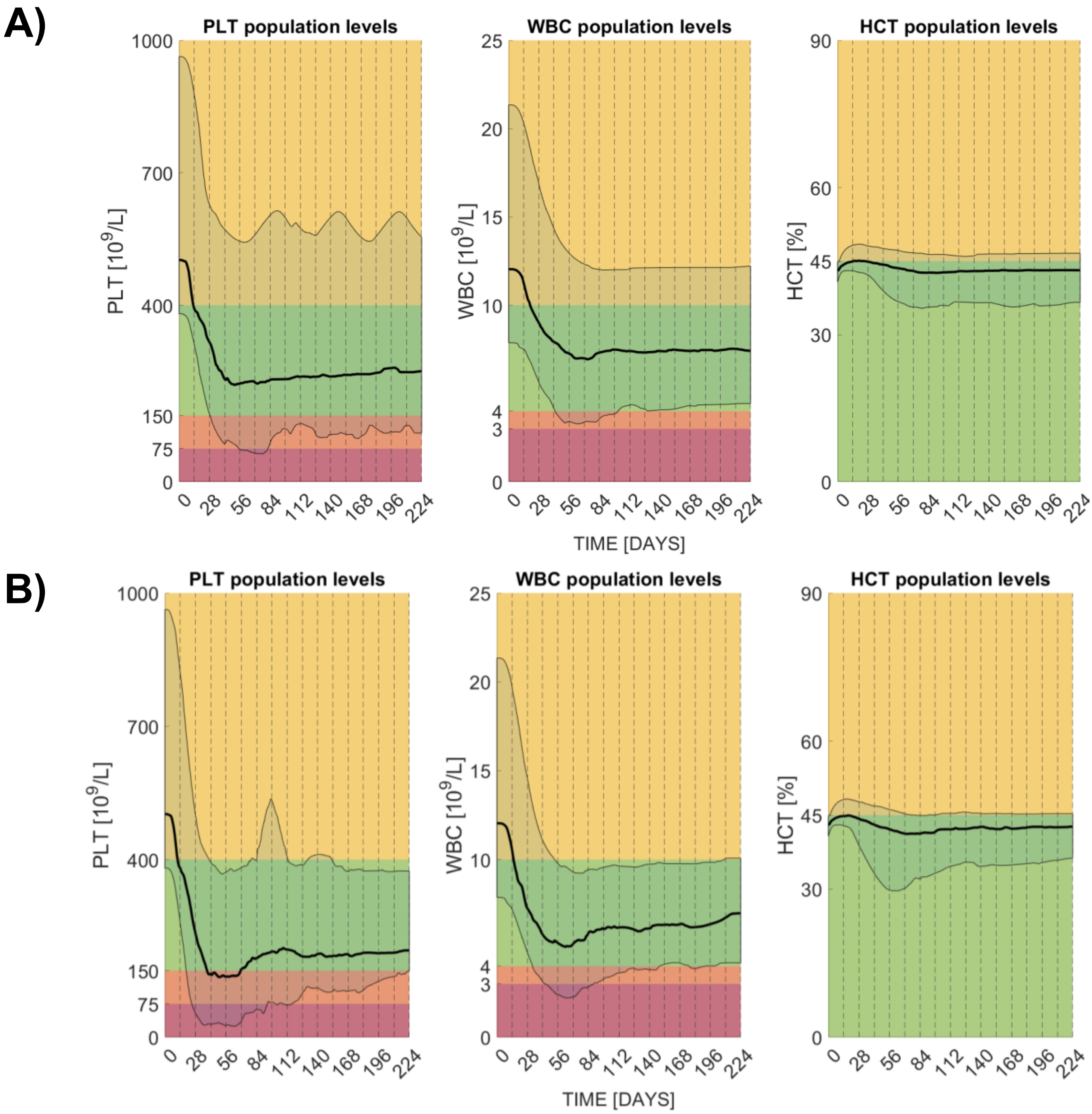

Figure S4. 1 Effect of Rew1 (Panel A) and Rew2 (Panel B) reward functions on the performances of the QL_{Pop}-agent. Results are summarized in terms of median (black line) and 90% C.I. of individual profiles within the population (blue shaded areas). Yellow, green, orange and red shaded areas, represents inefficacy, efficacy, moderate and severe toxicity ranges of each haematological parameter.

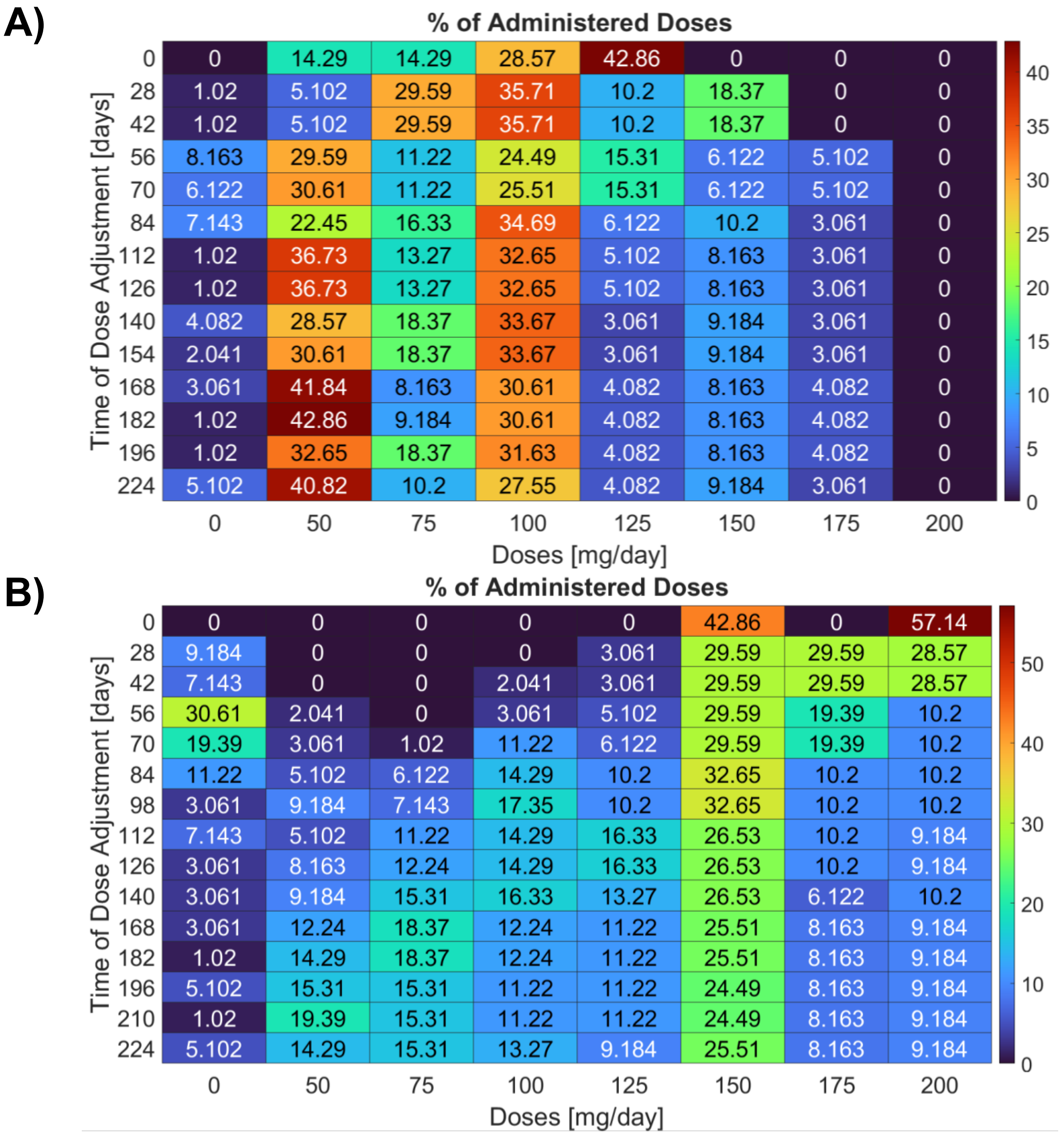

Figure S4. 2 Distribution of doses administered by the QL_{pop}-agents with Rew1 (Panel A) and Rew2 (Panel B) reward functions for each treatment cycle.
