# Supplementary material for: Precision Dosing in Presence of Multiobjective Therapies by Integrating Reinforcement Learning and PK‐PD Models: Application to Givinostat Treatment of Polycythemia Vera
Source: CPT Pharmacometrics Syst Pharmacol. 2025 May 5;14(6):1018–31. doi: 10.1002/psp4.70012 (PMC12167923; doi:10.1002/psp4.70012)
Supplement: Supplementary file 5 — Data S5. [file PSP4-14-1018-s004.pdf]

Supplementary Materials S5

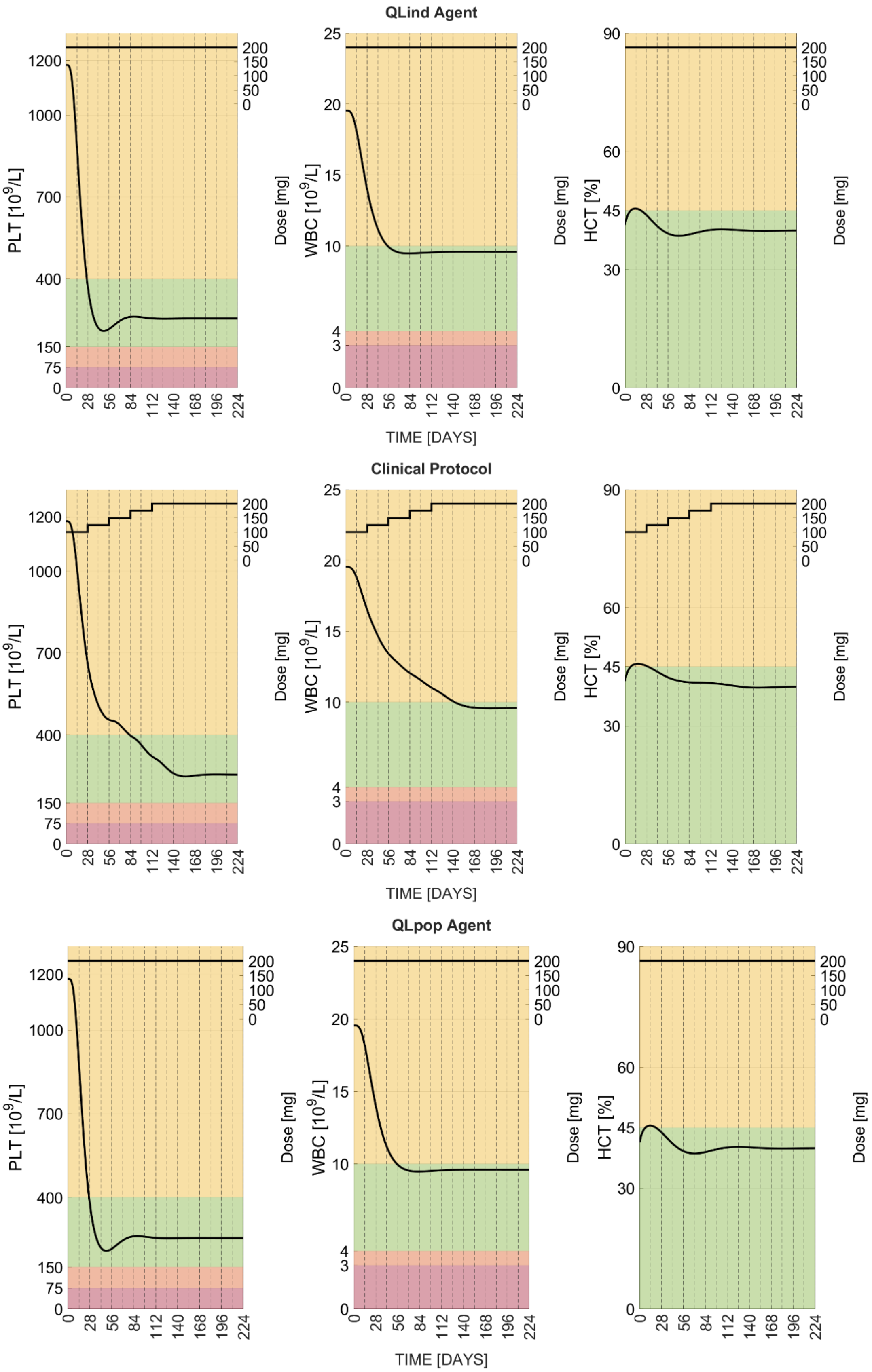

Figure S5. 1 Example of virtual PV patient on which the individual and population QL-agent outperform the clinical protocol by choosing the maximum givinostat dose from the beginning of the treatment. This strategy allows to quickly reach the CHR.

Yellow, green, orange and red shaded areas, represents inefficacy, efficacy, moderate and severe toxicity ranges of each haematological parameter.

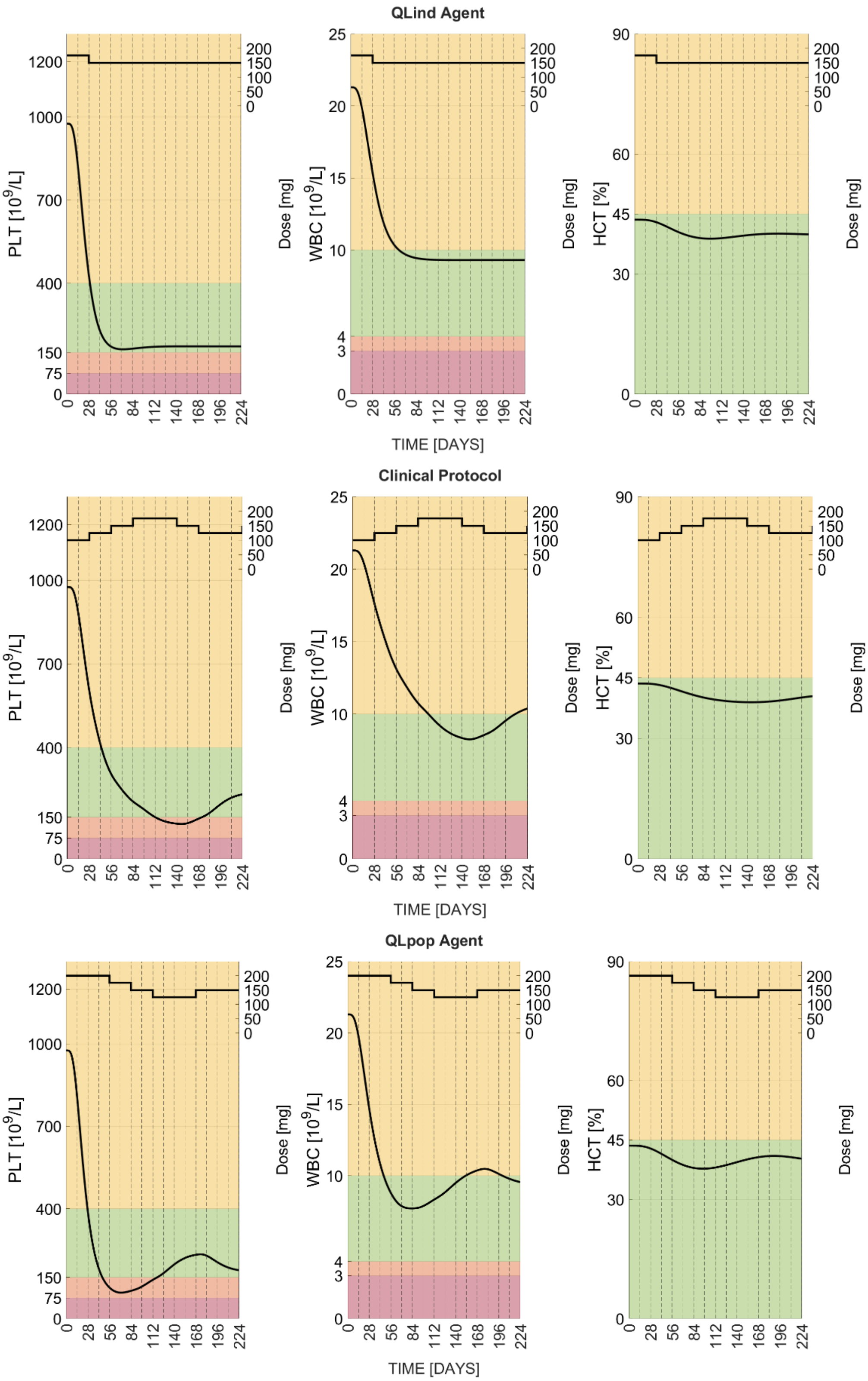

Figure S5. 2 Example of virtual PV patient on which the QL_{ind}-agent outperforms both the QL_{pop}-agent and clinical protocols by correctly choosing a loading dose which allows a quicker CHR. Yellow, green, orange and red shaded areas, represents inefficacy, efficacy, moderate and severe toxicity ranges of each haematological parameter.

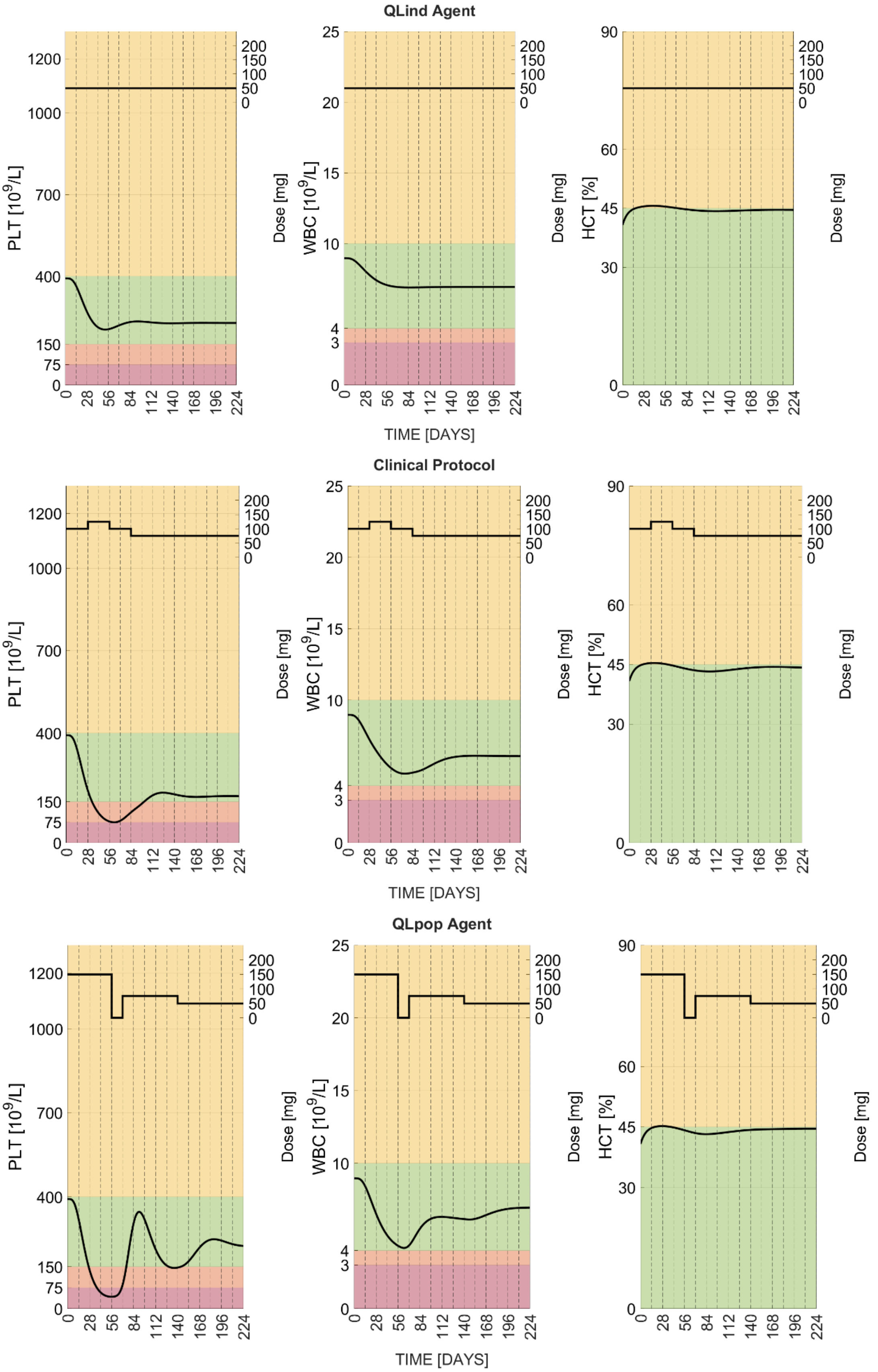

Figure S5. 3 Example of virtual PV patient on which the QL_{ind}-agent outperforms both the QL_{pop}-agent and clinical protocols by choosing the minimum givinostat dose from the beginning of the treatment. This strategy allows to reach the CHR avoiding toxicity events. Yellow, green, orange and red shaded areas, represents inefficacy, efficacy, moderate and severe toxicity ranges of each haematological parameter.

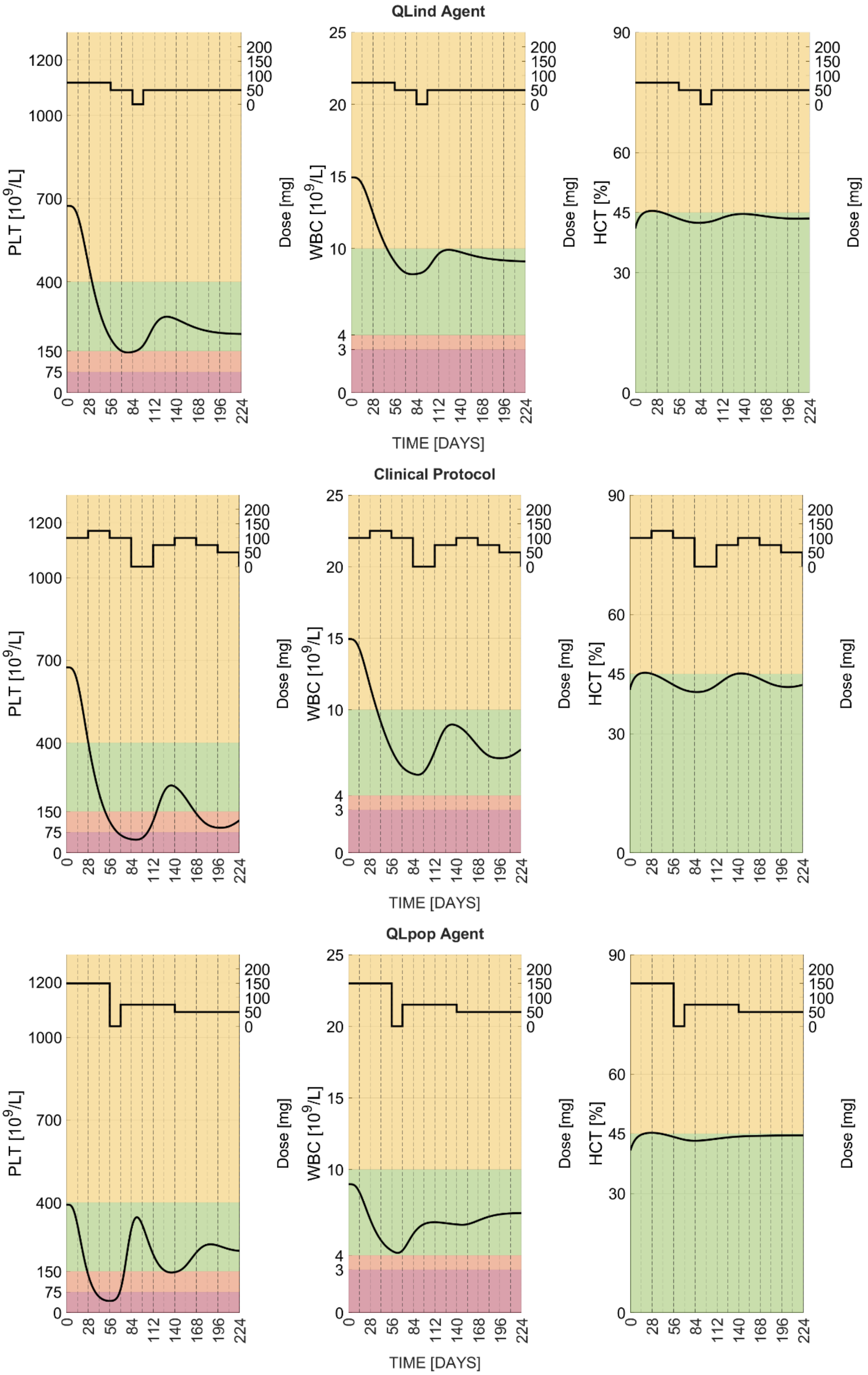

Figure S5. 4 Example of virtual PV patient on which the QL_{ind}-agent outperforms both the QL_{pop}-agent and clinical protocols with a dosing strategy which avoids the onset of severe toxicities. Yellow, green, orange and red shaded areas, represents inefficacy, efficacy, moderate and severe toxicity ranges of each haematological parameter.

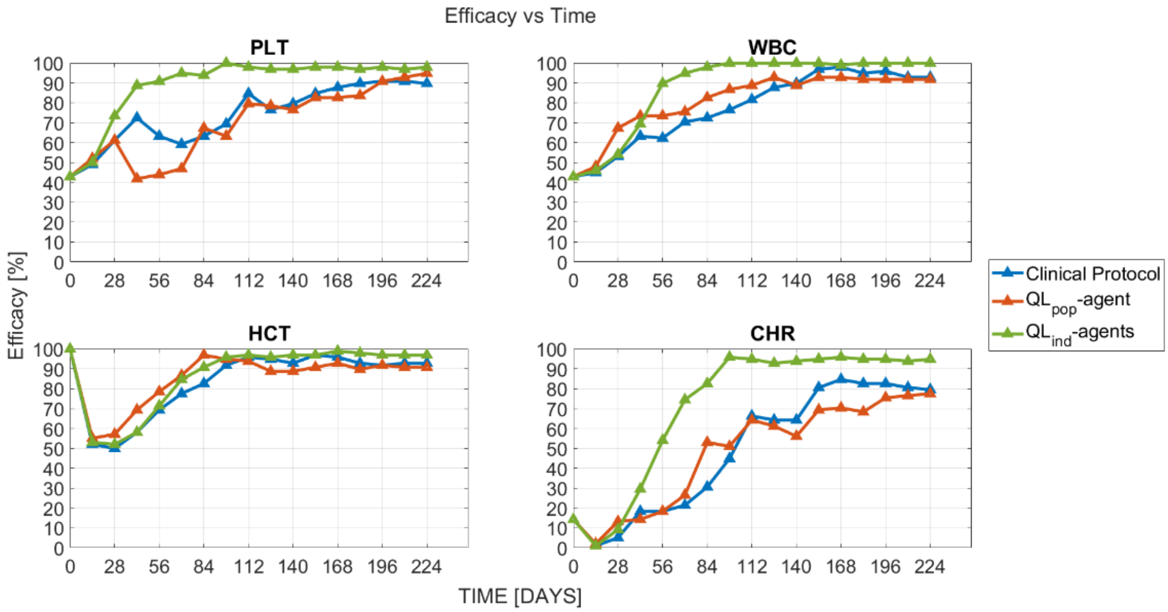

Figure S5.5: Comparison of efficacy rates over time for each haematological parameter and complete haematological response (CHR) across three treatment strategies: Clinical Protocol, QLpop-Agent and QLind-Agents.

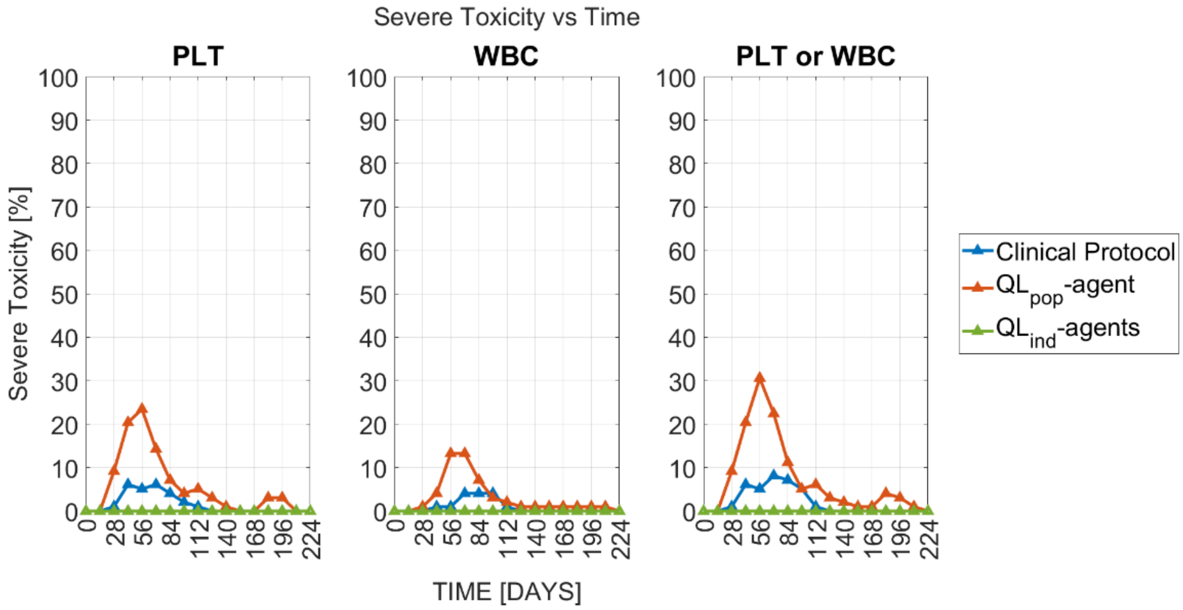

Figure S5.6: Comparison of severe toxicity rates over time for PLT, WBC and at least one of them (i.e., PLT or WBC) across three treatment strategies: Clinical Protocol, QLpop-Agent and QLind-Agents.

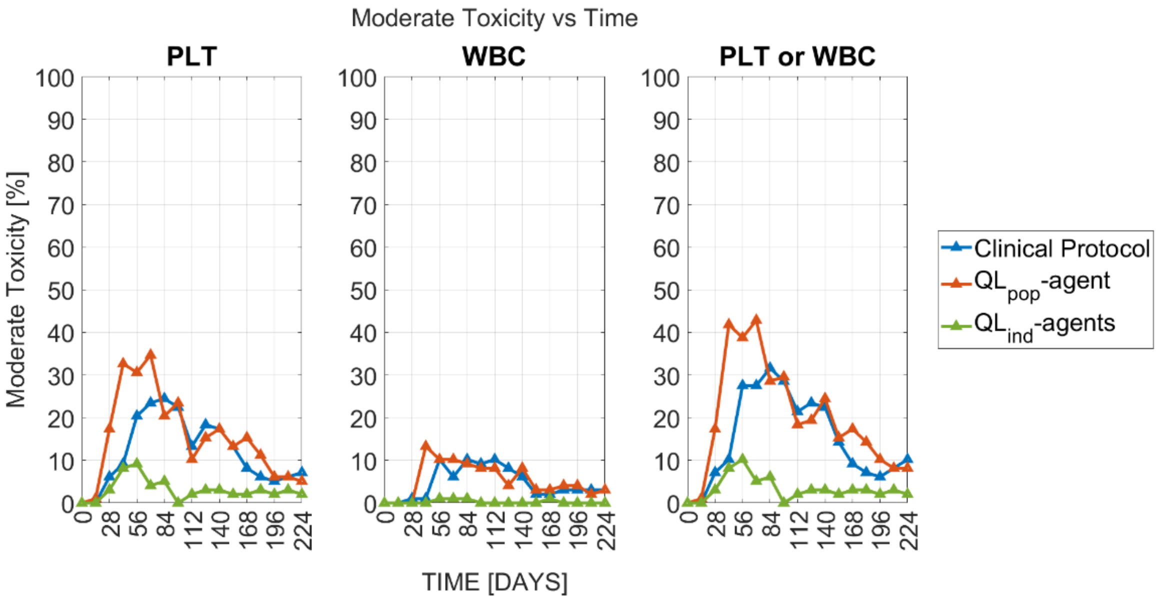

Figure S5.7: Comparison of moderate toxicity rates over time for PLT, WBC and at least one of them (i.e., PLT or WBC) across three treatment strategies: Clinical Protocol, QLpop-Agent and QLind-Agents.

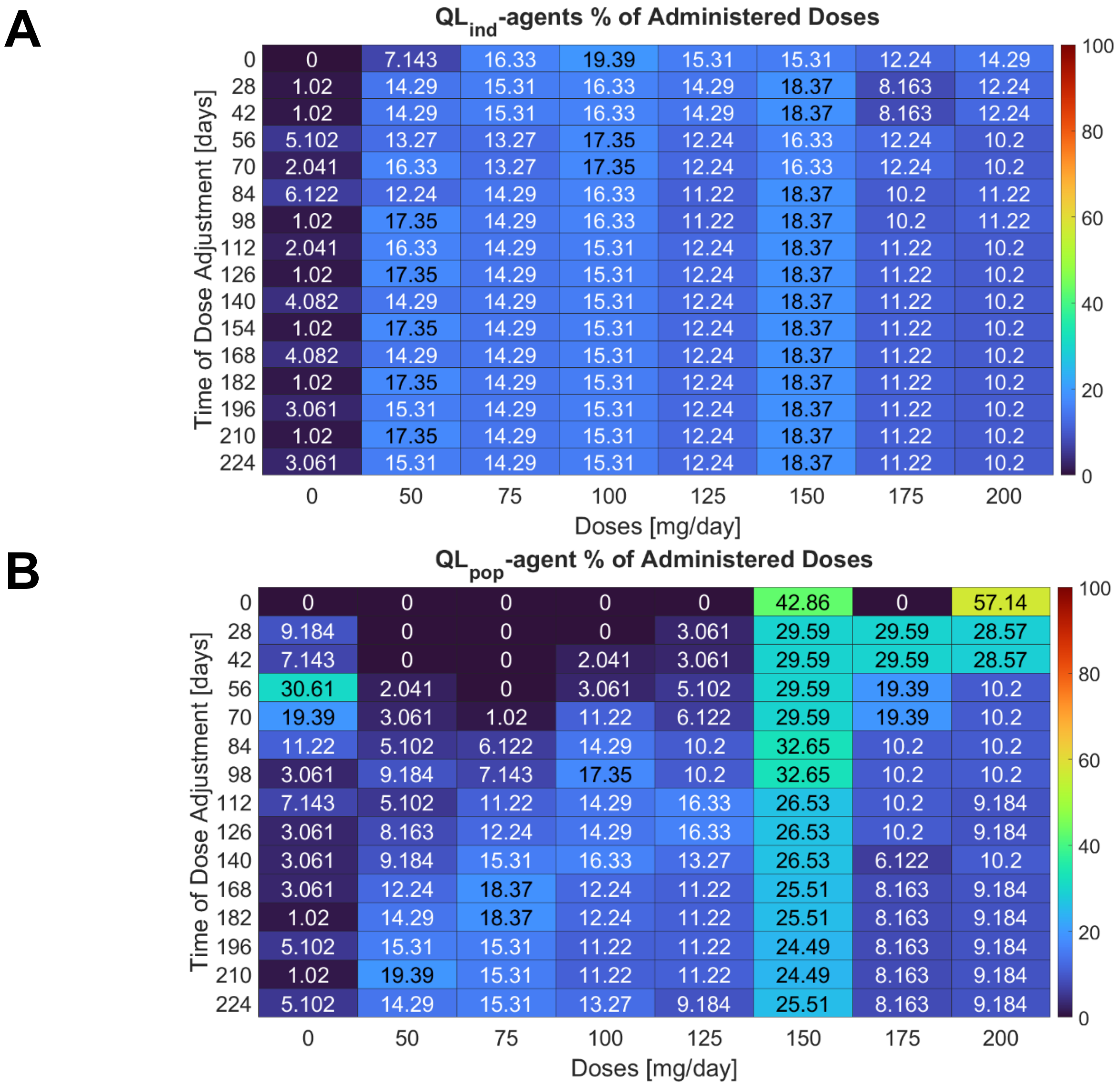

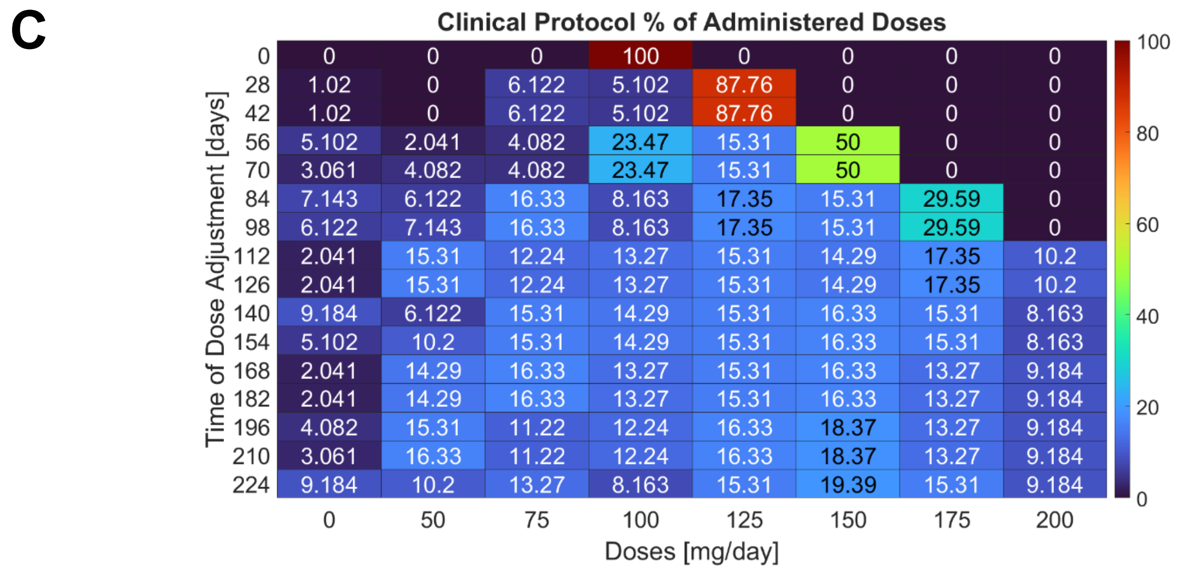

Figure S5.8: Distribution of the doses administered at each potential decisional step. A dose = 0 mg coincides with a temporary interruption of givinostat therapy. Panel A: QLind-agents, Panel B: QLpop-agent, Panel C: clinical protocol.
