# Supplementary material for: Precision Dosing in Presence of Multiobjective Therapies by Integrating Reinforcement Learning and PK‐PD Models: Application to Givinostat Treatment of Polycythemia Vera
Source: CPT Pharmacometrics Syst Pharmacol. 2025 May 5;14(6):1018–31. doi: 10.1002/psp4.70012 (PMC12167923; doi:10.1002/psp4.70012)
Supplement: Supplementary file 6 — Data S6. [file PSP4-14-1018-s005.pdf]

Supplementary Materials S6

The aim of this section is to provide a detailed description of how RL combined with PK-PD modelling can be used to individually optimize an adaptive dosing treatment within the context of clinical trials. In this case, the individual-oriented QL-approach was asked to tailor the givinostat treatment on each individual patient with the aim of maximizing the CHR rate at the eight month of treatment which is one of the primary endpoint of the planned phase III clinical study.

The formalization of the givinostat precision dosing problem described in the main text was adopted. Only the reward function used for QL_{ind}-agents (Eqs.1-11) was properly modified to emphasize the need of achieving a CHR at the end of the treatment (i.e., 8th month). In particular, as shown in Eq.S6.1 and Eq.S6.2, an additive bonus term was added to the reward function to renumerate more dosing actions able to induce a CHR at the end of the eighth month of treatment.

$$Reward = \begin{cases} 0 \quad if \ \ PLT_{Obs} < 75 \times 10^9/L \text{ and/or } WBC_{Obs} < 3 \times 10^9/L \\ Reward_{PLT} + Reward_{WBC} + Reward_{HCT} + \text{Bonus} \quad otherwise \end{cases}$$

(S6. 1)

$$Bonus = \begin{cases} 100 \quad if \ month = 8 \ and \ CHR \ is \ achieved \\ 0 \quad otherwise. \end{cases}$$

(S6. 2)

Individual QL-agents with bonus term ($QL_{ind\text{-}bonus}$-agents) were evaluated on the same virtual population used for the QL_{ind}-agents (i.e., those without bonus in the reward function). As shown in Table S6.1, $QL_{ind\text{-}bonus}$-agents were able to achieve the desired result, that is inducing a CHR in all the patients at the end of the treatment period. However, to ensure a CHR at that specific endpoint (i.e., end of eight month), $QL_{ind\text{-}bonus}$-agents slightly reduced treatment efficacy in the other months. Indeed, the $QL_{ind\text{-}bonus}$-agent policies ensured a shorter permanence of PLT, WBC and HCT in the efficacy ranges than the QL_{ind}-agents. Figure S6.1 better clarifies this difference between QL_{ind} (Panel A) and $QL_{ind\text{-}bonus}$ (Panel B) agents. In particular, for this patient, $QL_{ind\text{-}bonus}$ -agent prefers a dosing strategy leading to a longer PLT moderate toxicity (orange shaded area) in order to obtain CHR at the eight month. Conversely, QL_{ind}-agent, maximizes the permanence in the target range (green shaded area) though CHR is not achieved at eighth month.

Table S6. 1 Comparison between QLind Agents and QLind-bonus-agents on the same virtual population of 98 patients. For each metric, the median and 95% C.I. in the population are reported.

**QL_{ind}-agents**	**$QL_{ind\text{-}bonus}$-agents**
**% of Response on 8 months of treatment – PLT**	
96.90	100
**% of Response on 8 months of treatment – WBC**	
100	100
**% of Response on 8 months of treatment – HCT**	
95.90	100
**% of Response on 8 months of treatment – CHR**	
93.80	100
**% of Days on 8-months treatment with severe Toxicity**	
0	0
[0,0]	[0,0]
**% of Days on 8-months treatment with $PLT \in [150, 400] \times 10^9/L$ [95% C.I]**	

91.56	90.44
[41.67,100]	[43.97,100]
**% of Days on 8-months treatment with WBC$\in [4, 10] \times 10^9/L$ [95% C.I]**	
88.44	87.33
[64.8,100]	[61.04,100]
**% of Days on 8-months treatment with HCT <45% [95% C.I]**	
94.00	93.11
[46.13,100]	[50.49,100]
**% of Days on 8-months treatment with CHR [95% C.I]**	
75.56	73.33
[33.44,88.89]	[27.09,88.89]

A)

B)

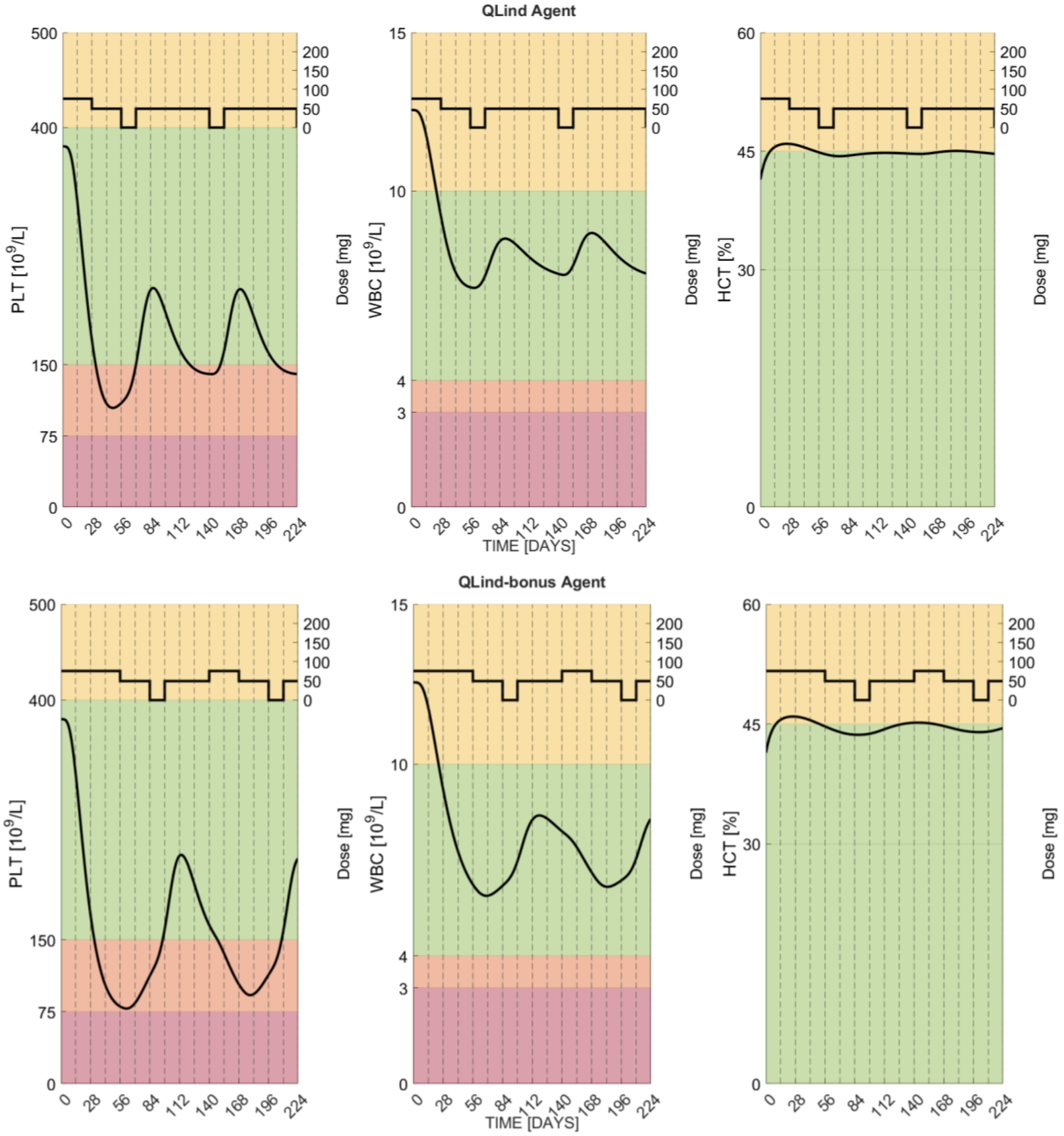

Figure S6. 1 An example of how the introduction of bonus term in the reward function affects QL treatment personalization. For the same patient, Panel A shows the dosing strategy of QL_{ind}-agent (i.e., without bonus), conversely, Panel B illustrates the strategy proposed by $QL_{ind-bonus}$-agent. Yellow, green, orange and red shaded areas, represents inefficacy, efficacy, moderate and severe toxicity ranges of each haematological parameter.
