# Supplementary material for: Precision Dosing in Presence of Multiobjective Therapies by Integrating Reinforcement Learning and PK‐PD Models: Application to Givinostat Treatment of Polycythemia Vera
Source: CPT Pharmacometrics Syst Pharmacol. 2025 May 5;14(6):1018–31. doi: 10.1002/psp4.70012 (PMC12167923; doi:10.1002/psp4.70012)
Supplement: Supplementary file 7 — Data S7. [file PSP4-14-1018-s001.pdf]

Supplementary Materials S7

Evaluating the differences between QL_{ind} protocols

The aim of this section is to quantitatively evaluate the similarity between the dosing rules estimated for each patient by QL_{ind}-agents. To this end, first, we converted the set of personalized rules estimated by QL_{ind}-agents into 487-dimensional numerical vectors in which each entry represents a patient state and corresponds to the dose suggested by the QL algorithm for that state. In this way, we obtained 98 vectors, one for each virtual patient. Then, we computed the Euclidean distance (Eq. S7.1) between each pair of vectors (i.e., pair of patients).

$$d(\boldsymbol{X_1}, \boldsymbol{X_2}) = \sqrt{\sum_{i=1}^{487} \left(X_{1,i} - X_{2,i}\right)^2}$$

(S7.1)

Considering the patient stratification defined by the steady-state analysis of the givinostat PK-PD model, for each cluster $c_i$we computed the intra-cluster distance (ICD), i.e., the median distance among patients belonging c_i, and the between-clusters distances (BCDs), i.e., the median distances between patients in c_i and patients in another cluster, c_j.

Figure S7.1**Errore. L'origine riferimento non è stata trovata.** illustrates the ICDs and BCDs computed for each of the 14 clusters defined based on the response pattern at steady-state. Panel A reports ICDs and BCDs values for the response pattern clusters including only one optimal givinostat dose level (i.e., <50, 50, 75, 100, 125, 150, 175, 200 mg). Distances between contiguous response pattern clusters (e.g., 50 and <50 or 200 and 175 mg) were significantly lower than distance between very different response patterns (i.e., 50 and 200 mg), thus confirming that the QL-based protocols are able to capture these differences in terms of response. This finding occurs also in panel B, which focuses on showing ICDs and BCDs of response pattern clusters characterized by two optimal givinostat dose levels (e.g., 50 and 75 mg, 100 and 125 mg). Finally, panel C of Figure R.4 shows the BCDs among response clusters characterized by one and two givnostat dose level, respectively. Here, it can be observed that the clusters with two doses are closer to the clusters containing one of the two or contiguous dose levels.

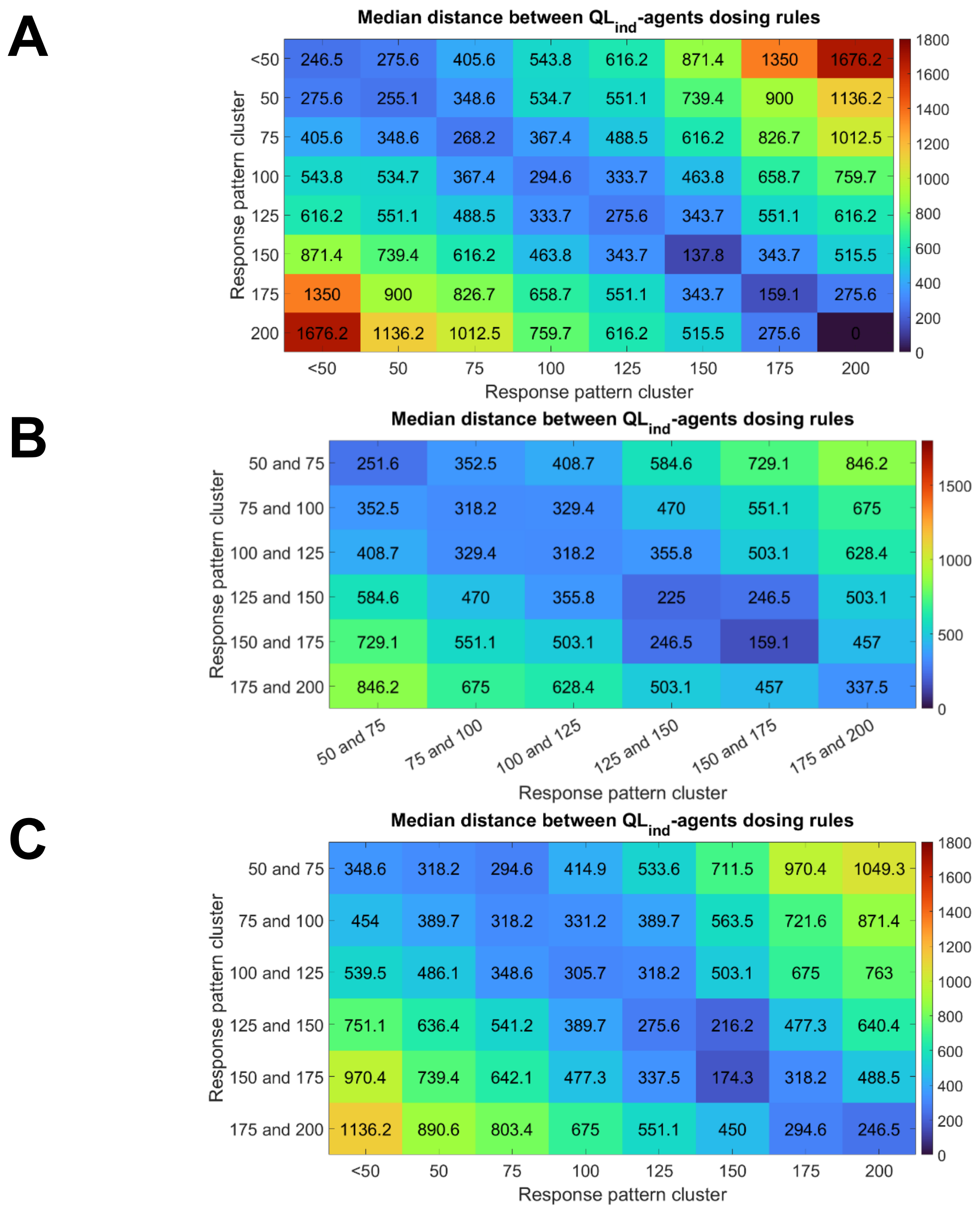

Figure S7.1: ICDs and BCDs among the different givinostat response patterns. Panel A focuses on ICDs and BCDs values for the response pattern clusters including only one optimal givinostat dose level. Panel B reports ICDs and BCDs computed among response pattern clusters having two optimal givinostat doses. BCDs among response clusters characterized by one and two givnostat dose level, respectively.
